# Inhibiting IP6K1 confers atheroprotection by elevating circulating apolipoprotein A-I

**DOI:** 10.1016/j.metabol.2024.156098

**Published:** 2024-12-04

**Authors:** Xiaoqi Liu, Zixuan Zhang, Tim Aguirre, Megan L. Shipton, Lin Fu, Jimin Du, David Furkert, Ji Qi, Alfred C. Chin, Andrew M. Riley, Tong Liu, Xu Zhang, Barry V. L. Potter, Dorothea Fiedler, Yi Zhu, Chenglai Fu

**Affiliations:** 1 Tianjin Key Laboratory of Metabolic Diseases, Department of Physiology and Pathophysiology, The Province and Ministry Co-Sponsored Collaborative Innovation Center for Medical Epigenetics, Tianjin Medical University, 22 Qixiangtai Road, Tianjin, 300070, China; 2 Leibniz-Forschungsinstitut für Molekulare Pharmakologie (FMP), Berlin, 13125, Germany; 3 Medicinal Chemistry & Drug Discovery, Department of Pharmacology, University of Oxford, Mansfield Road, Oxford OX1 3QT, UK; 4 Tianjin Key Laboratory of Ionic-Molecular Function of Cardiovascular Disease, Department of Cardiology, Tianjin Institute of Cardiology, Second Hospital of Tianjin Medical University, Tianjin, 300211, China; 5 Institute for Developmental and Regenerative Cardiovascular Medicine, Xinhua Hospital, School of Medicine, Shanghai Jiao Tong University, Shanghai 200092, China; 6 Weill Cornell/Rockefeller/Sloan Kettering Tri-Institutional MD-PhD Program, New York, NY, 10065, USA

**Keywords:** IP6K, HDL, cholesterol efflux, UBE4A, atherosclerosis, hepatocyte, inositol pyrophosphate

## Abstract

**Background and aims:**

Atherosclerotic cardiovascular diseases are the leading cause of death. Apolipoprotein A-I (apoA-I) mediates cholesterol efflux to lower the risks of atherosclerosis. Elevating circulating apoA-I is an effective strategy for atheroprotection. However, the regulatory mechanisms of apoA-I have been elusive.

**Methods:**

Protein-protein interactions were examined by co-immunoprecipitations. Chemical biology tools were used to determine the binding of 5PP-InsP_5_ to its target proteins and its roles in mediating protein-protein interactions. The mouse atherosclerotic model was generated by injecting AAV-PCSK9 and feeding a Western diet. Atherosclerotic plaques were determined by Oil Red O and H&E staining.

**Results:**

We show that blocking IP6K1 activity increases apoA-I production in hepatocytes. IP6K1 binds to apoA-I and via its product 5PP-InsP_5_ to induce apoA-I degradation, which requires ubiquitination factor E4A (UBE4A). Depleting 5PP-InsP_5_ by deleting IP6K1 or blocking IP6K1 activity disrupts the interaction between UBE4A and apoA-I, preventing apoA-I degradation, leading to increased production of apoA-I. Hepatocyte-specific deletion of IP6K1 elevates circulating apoA-I levels, which augments cholesterol efflux and lowers the burden of atherosclerosis. Mice with both *apoA-I* KO and hepatocyte-specific *IP6K1* KO were generated to validate that IP6K1 deletion-induced atheroprotection requires apoA-I.

**Conclusions:**

Our findings reveal a mechanism by which blocking IP6K1 boosts apoA-I production. Blocking IP6K1 represents a potential treatment strategy to elevate circulating apoA-I for atheroprotection.

## Graphical abstract

**Graphical abstract:**
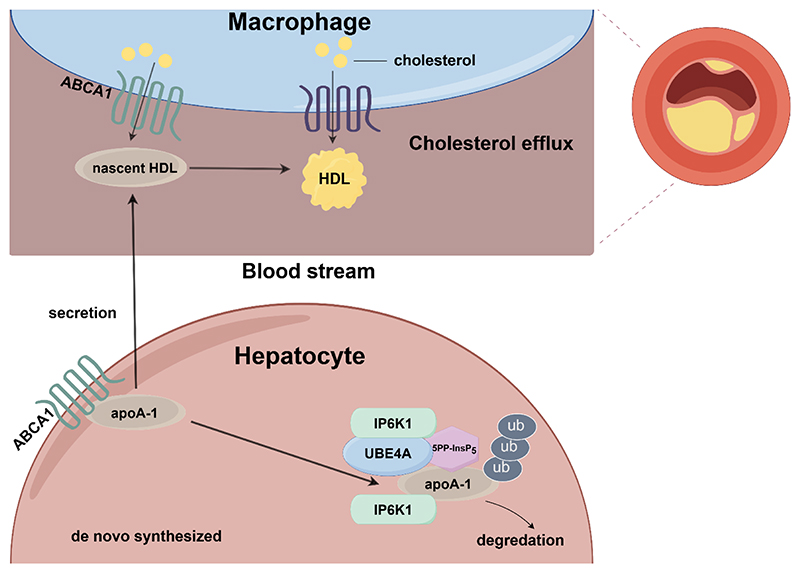

## 1 Introduction

Cardiovascular diseases associated with atherosclerosis are the leading cause of death worldwide. Cholesterol accumulation in the blood vessels is a major contributor to the development of atherosclerosis. High-density lipoprotein (HDL) removes cholesterol from arteries [1]. Enhancing HDL-mediated reverse cholesterol transport represents a promising strategy for atherosclerosis treatment [2–5]. Apolipoprotein A-I (apoA-I) is the major structural and functional HDL protein, accounting for approximately 70 % of total HDL proteins [6]. Newly synthesized apoA-I acquires cholesterol and phospholipids to form nascent HDL[7]. Preclinical studies show that upregulating endogenous apoA-I, transgenic overexpression or exogenous application of apoA-I enhances reverse cholesterol transport, and decreases atherosclerotic plaque lesion size [8–11]. Clinical trials demonstrate that administration of apoA-I to patients with coronary artery disease lowers atherosclerosis burden[4, 12]. However, the regulatory mechanisms of apoA-I have been elusive.

Inositol hexakisphosphate kinase 1 (IP6K1) is ubiquitously expressed in mammalian cells. It generates 5-diphosphoinositol pentakisphosphate (5PP-InsP_5_) to mediate diverse functions [13–15]. Preclinical studies indicate that IP6K1 is a promising therapeutic target for diabetes and obesity[16–18]. Furthermore, blocking 5PP-InsP_5_ biosynthesis by deleing IP6K1 also protects the heart against ischemia-reperfusion injury [19]. However, the roles of IP6K1 and its derived 5PP-InsP_5_ in metabolic dysfunction-related cardiovascular diseases have not been demonstrated.

Ubiquitination factor E4A (UBE4A) is a widely expressed E3 ubiquitin ligase. It participates in multiple physiological processes [20–25], and is required for normal development [22]. On the other hand, deletion of UBE4A stabilizes p53, inhibiting cancer cell proliferation by preventing UBE4A-induced p53 degradation[23]. Targeting UBE4A can also enhance the host’s antiviral defense[24].

In this study we found that IP6K1, via its product 5PP-InsP_5_, promotes UBE4A-dependent apoA-I degradation in hepatocytes. Depleting 5PP-InsP_5_ by deleting IP6K1 or pharmacological inhibiting IP6K1 prevents UBE4A-mediated apoA-I degradation and thus increases apoA-I production. Hepatocyte-specific deletion of IP6K1 elevates circulating apoA-I, leading to enhanced cholesterol efflux and reduced burden of atherosclerosis. We generated the animals with both *apoA-I* KO and hepatocyte-specific *IP6K1* KO to demonstrate that deletion of IP6K1 confers atheroprotection by elevating circulating apoA-I levels.

## 2 Material and methods

### 2.1 Cell culture and transfection

HEK 293 cells, HEK 293T cells, and murine macrophage J774A.1 cells were cultured in Dulbecco’s Modified Eagle Medium (DMEM) (Thermo Fisher Scientific) supplemented with 10% (v/v) fetal bovine serum (Thermo Fisher Scientific), penicillin (100 U/mL), and streptomycin (100 μg/mL) (Thermo Fisher Scientific). Murine AML12 hepatocytes were cultured in DMEM/F-12 (Thermo Fisher Scientific) supplemented with 10% (v/v) fetal bovine serum (Thermo Fisher Scientific), 1% (v/v) ITS Liquid Media (Sigma-Aldrich), Dexamethasone (40 ng/mL) (Solarbio), penicillin (100 U/mL), and streptomycin (100 μg/mL) (Thermo Fisher Scientific). Primary mouse hepatocytes of wild type (WT) and *IP6K1* knockout (KO), and primary mouse hepatocytes of *IP6K1*^flox/flox^ and *Alb*-cre; *IP6K1*^flox/flox^ were cultured in RPMI 1640 medium (Thermo Fisher Scientific) supplemented with 10% (v/v) fetal bovine serum (Thermo Fisher Scientific), penicillin (100 U/mL), and streptomycin (100 μg/mL) (Thermo Fisher Scientific). All cells were maintained at 37°C with 5% CO_2_. Cells were plated one day before the experiments. Before treating cells, the existing cell culture medium was exchanged with fresh medium. Transfections were conducted with Lipofectamine 3000 (Thermo Fisher Scientific). PPIP5K1-deleted murine AML12 hepatocytes, PPIP5K2-deleted murine AML12 hepatocytes and UBE4A-deleted murine AML12 hepatocytes were generated by shRNA transduction. *N*^2^-(*m*-(trifluoromethyl)benzyl) *N*^6^-(*p*-nitrobenzyl)purine (TNP), an inhibitor of IP6K, was purchased from MilliporeSigma. MG132 (S2619) was purchased from Selleck Chemicals.

### 2.2 Animal experiments

WT and *IP6K1* KO mice were littermates from heterozygous breeding. Hepatocyte-specific *IP6K1* KO mice (*Alb*-cre; *IP6K1*^flox/flox^) were generated by crossing *Alb*-cre strain with *IP6K1*^flox/flox^ strain. *ApoA-I* KO mice were purchased from The Shanghai Model Organisms Center, Inc. The *Alb*-cre; *IP6K1*^flox/flox^; *apoA-I* KO mice were generated by crossing *apoA-I* KO mice with the *Alb*-cre; *IP6K1*^flox/flox^ mice. Male animals from the same litter, aged 6-8 weeks, were randomly assigned to experimental groups. In order to follow the 3R principles, and not affect the power of the experimental test, 3-6 mice per group were considered necessary.

Blood collection: Animals were euthanized by using CO_2_, blood was collected via cardiac puncture, heparin was used as anti-coagulant. Plasma was separated by centrifugation. Plasma levels of high-density lipoprotein cholesterol (HDL-C), low-density lipoprotein cholesterol (LDL-C), total cholesterol, and total triglycerides were determined by using kits (BioSino Bio-Technology and Science, Beijing, China), following manufacturer’s protocols.

Atherosclerotic model: The concentrated stocks of adeno-associated virus expressing mouse recombinant PCSK9 with gain-of-function mutation D377Y (AAV-PCSK9) were purchased from the Vigene Biosciences (Shandong, China). Mice were injected via the tail vein with 100 μL sterile PBS containing 1×10^11^ viral particles of AAV-PCSK9, and were fed with a Western diet (TD88137, Harlan) for 12 weeks to induce atherosclerosis. The animal was included in the study if the expression of LDLR in the liver was markedly knocked down and the total cholesterol in the blood plasma was significantly increased. No animal was excluded in this experiment.

Imaging and analysis of Oil Red O-stained whole aorta lesions were performed as previously reported[26]. Mice were euthanized by intraperitoneal injection of ketamine and xylazine, then were perfused with PBS. The aorta and its branches were dissected and adventitial adipose and connective tissue were removed. The aorta was fixed with 4% paraformaldehyde dissolved in PBS through apical left ventricular puncture. The whole aorta was excised, cut open longitudinally and the adipose tissue was cleaned carefully. The whole aorta was pinned onto a wax Petri dish, and then stained with Oil Red O for 1 hour at room temperature. The aorta was then washed once for 20 min with 60% isopropanol at room temperature, then rinsed three times with distilled H_2_O for 5 min to remove isopropanol. An image of the whole aorta was captured.

Staining of the aortic root: Mice were euthanized by intraperitoneal injection of ketamine and xylazine, perfused with PBS, and fixed with 4% (w/v) paraformaldehyde. The aortic root from the heart was embedded in OCT and then serially sectioned. Sections containing three aortic valves per mouse were stained with H&E and Oil Red O for total lesion, necrotic core, and lipid accumulation area quantification. The necrotic core areas were the areas without cellular components in the plaques.

TNP was dissolved in dimethyl sulfoxide (DMSO): Tween 80: water (1:1:8), and was injected intraperitoneally (20 mg/kg; body weight) once a day for one week[27]. SC-919 was dissolved in 0.5% (w/v) methylcellulose solution, and was orally administered (10 mg/kg) once a day for one week[18].

Animal breeding and procedures were conducted in accordance with the NIH Guide for Care and Use of Laboratory Animals and approved by the Animal Care and Use Committee of Tianjin Medical University. The approval number for animal experiments is TMUaMEC2023023. All mice were bred on a C57BL/6 background and maintained under a 12:12 hours light/dark cycle (lights on at 7:00 and off at 19:00) before and during experiments. Three investigators participated in the animal studies: a first investigator (Xiaoqi Liu) conducted the experiment, a second investigator (Jimin Du) was responsible for the outcome assessment, and a third investigator (Zixuan Zhang) analyzed the data.

### 2.3 Enzyme-linked immunosorbent assay

Animals were euthanized by CO_2_. Blood samples were collected via cardiac puncture. The plasma concentrations of apoA-I were determined by using a mouse apoA-I ELISA kit, following the manufacturer’s instructions (E-EL-M3016, Elabscience).

### 2.4 Isolation of primary mouse hepatocytes

Primary mouse hepatocytes were isolated as previously reported[28]. Mice were anesthetized by intraperitoneal injection of ketamine/xylazine. The inferior vena cava was cannulated and the liver was perfused by using Hank’s Balanced Salt Solution containing 0.5 mM EDTA. The liver was then digested by perfusing with Hank’s Balanced Salt Solution containing 0.01% collagenase (w/v) and 2 mM CaCl_2_. The liver was dissected out, and the hepatocytes were gently released and filtered through a 70 μm cell strainer into a 50 mL tube. After centrifugation at 50 g for 2 min, the cell pellet was re-suspended with RPMI 1640 medium. An equal volume of Percoll solution was added to the cell solution and mixed thoroughly then spun at 200 g for 10 min to remove the dead cells and debris. The cell pellet of viable hepatocytes was re-suspended with RPMI 1640 medium and plated on collagen-coated cell culture plates. The cell culture media was exchanged to fresh media 4 hours later, after the cells had attached to the culture plate.

### 2.5 Cellular cholesterol efflux assay

The cellular cholesterol efflux assay was performed by using a cholesterol efflux assay kit (ab196985; Abcam), following the manufacturer’s protocol. Briefly, the macrophage J774A.1 cells were attached to the plates, the cell monolayer was washed with RPMI 1640 media (no serum added), and then labeled with fluorescent cholesterol for 16 hours in a humidified incubator at 37°C protected from light. The labeling reagent was removed and the cells were washed with RPMI media (no serum). Mice plasma (depleted LDL/VLDL) were diluted in RPMI media (no serum), added to the cells, and then incubated for 4 hours in a 37°C incubator. The supernatants were collected and the fluorescence was measured (Ex/Em = 482/515 nm). The cell monolayer was solubilized by using cell lysis buffer and the fluorescence was measured (Ex/Em = 482/515 nm). Cellular cholesterol efflux was calculated by dividing the fluorescence intensity of the media by the total fluorescence intensity of the cell lysate of the same treatment and media. $\;\%\;Cellular\;cholesterol\;efflux=\frac{\;Fluorescence\;Intensity\;of\;Media\;}{\;Fluorescence\;Intensity\;of\;Cell\;Lysate\;+\;Media\;}\times 100$.

### 2.6 Western blots and immunoprecipitation

Tissues and cells were homogenized by pestle at 4ºC in lysis buffer containing 50 mM Tris-HCl (pH 7.4), 100 mM NaCl, 0.5% Igepal CA630, 5 mM MgCl_2_ and protease/phosphatase inhibitors (Roche, Switzerland). Lysates were pulse sonicated and centrifuged at 14,000 g for 10 min at 4ºC. The supernatants were collected. Protein concentrations were normalized using a Pierce BCA Protein Assay Kit (Thermo Fisher). SDS loading buffer containing 5% β-mercaptoethanol was added and the samples were boiled for 5 min.

For immunoprecipitation, tissues and cells were homogenized by pestle at 4°C in a lysis buffer containing 50 mM Tris-HCl (pH 7.4), 100 mM NaCl, 0.5% Igepal CA630, 5 mM MgCl_2_ and protease/phosphatase inhibitors (Roche, Switzerland). Lysates were passed through 30-gauge needles 20 times and centrifuged at 14,000 g for 10 min at 4°C. The supernatants were collected and precleaned with protein A/G beads (Santa Cruz Biotechnology) for 90 min at 4°C. Lysates were centrifuged briefly, and the supernatants were collected while the protein A/G beads were discarded. Primary antibody (2μg/sample) was added to cell lysates and incubated at 4°C overnight. Protein A/G beads were then added to the cell lysates and incubated for 2 hours at 4°C. The beads were washed with cold lysis buffer three times. SDS loading buffer (1.5×) containing 5% β-mercaptoethanol was added and the samples were boiled for 5 min.

Samples were run on 10% polyacrylamide gels and transferred to nitrocellulose membranes (Thermo Fisher Scientific). Membranes were blocked with 5% milk in TBST for 30 min at room temperature, washed with TBST, and incubated with primary antibodies (1:1000 dilution) in 5% BSA in TBST overnight at 4ºC. The following day, membranes were washed with TBST, incubated with secondary antibodies (1:5000 dilution) for 1 hour at room temperature, and washed again with TBST. Chemiluminescent substrate (Thermo Fisher Scientific) was used to visualize protein bands.

Antibodies against IP6K1 (sc-374292), UBE4A (sc-365904), myc-tag (clone 9E10), apoA-I (sc-58230), GRP78 (sc-166490), Lamin (sc-71481), apoE (sc-390925), apoA-V (393722) and apoB (sc-393636) were purchased from Santa Cruz Biotechnology. Antibodies against β-actin (Cat No.66009), apoA-I (Cat No.14427-1-AP), GST (Cat No.100-0-AP) and ABCG1 (Cat No.13578-1-AP) were purchased from Proteintech. Antibodies against ubiquitin (3936s), pan-cadherin (4068s), ABCA1 (96292s) and O-GlcNAc (9875s) were purchased from Cell Signaling Technology. Antibody against flag-tag (MAB3118) was purchased from Millipore Sigma. Antibody against PPIP5K1 (A-304-920A-T) and PPIP5K2 (A-304-167A-T) was purchased from Thermo Fisher. Antibody against apoA-I (a1129) and LDLR (A14996) was purchased from ABclonal. Antibody against HA-tag (901501) was purchased from Biolegend. Antibody against albumin (ab207327) was purchased from abcam.

### 2.7 RNA isolation and real-time PCR

Total RNA was isolated from mice tissues and cells using the RNeasy Mini Kit (Qiagen) and reverse transcribed to complementary DNA using SuperScript III (Invitrogen), following the manufacturer’s recommended protocol. Real-time quantitative PCR was performed with the Brilliant II SYBR Green qPCR Master Mix (Stratagene) and the ABI 7900HT Real-time PCR System (Life Technologies). β-actin was used as loading control.

### 2.8 Subcellular fractionations

The endoplasmic reticulum (ER) was isolated by using a kit (Sigma-Aldrich ER0100), following the manufacturer’s instructions. The membrane and cytosol fractions were isolated by using an extraction kit (Beyotime, P0033), following the manufacturer’s instructions.

### 2.9 Immuno-electron microscopy

Mice were euthanized by intraperitoneal injection of ketamine and xylazine, then perfused and fixed with buffer containing 4% (w/v) paraformaldehyde, 0.2% (w/v) glutaraldehyde, 3% (w/v) Sucrose, 3 mM MgCl_2_ in 0.1 M phosphate buffer (pH 7.4). The livers were excised and a 1 mm^3^ of tissue was cut and fixed overnight at 4°C. The samples were then washed with pre-cooled 0.1 M phosphate buffer (pH 7.4) three times. The samples were dehydrated using a series of graded ethanol solutions (from 30% to 100% ethanol), and then embedded in LR white resin. Sections were cut 80 nm thick. Samples were blocked with 1% BSA solution for 30 min at room temperature after being washed three times with TBST and incubated in the primary antibodies (apoA-I: 14427-1-AP from Proteintech, IP6K1: sc-376290 from Santa Cruz Biotechnology, UBE4A: sc-365904 from Santa Cruz Biotechnology) diluted (1:50 dilution) in 1% BSA solution overnight at 4°C. The samples were then washed three times with TBST and incubated with secondary antibodies (12 nm Colloidal Gold AffiniPure™ Goat Anti-Rabbit IgG: 111-205-144 from Jackson ImmunoResearch, 4 nm Colloidal Gold AffiniPure™ Goat Anti-Mouse IgG: 115-185-146 from Jackson ImmunoResearch) diluted (1:25 dilution) in 1% BSA solution for 1 hour at room temperature followed by five washes with TBST and five washes with ultra-pure water. The samples were stained with 2% uranium acetate for 15 min with avoidance of light, rinsed in 70% ethanol three times and then rinsed in ultra-pure water three times. The samples were then dried at 37°C in an oven for 10 min. Images were taken under an electronic microscope.

### 2.10 Plasmid cloning

Myc-tagged WT IP6K1, myc-tagged kinase defective mutant (mut) IP6K1, myc-tagged GFP, flag-tagged apoA-I, flag-tagged GFP, HA-tagged UBE4A, GST-fused apoA-I, and GST-fused UBE4A were cloned into the pCDH-EF1-MCS-T2A-copGFP vector (System Biosciences). The PCR products were generated by using Phusion Polymerase (Thermo Fisher Scientific) and inserted into vectors using In-Fusion HD Enzyme (Takara Bio). IP6K1 shRNAs UBE4A shRNAs, PPIP5K1 shRNAs, and PPIP5K2 shRNAs were purchased from MilliporeSigma. All newly constructed plasmids were sequence-verified.

### 2.11 Lentivirus generation

HEK 293T cells were plated one day before experiments and allowed to grow to 70% confluence. Lentiviral vectors harboring gene of interest together with pMD2.G and psPAX2 were transfected into HEK 293T cells using Lipofectamine 3000. Cell culture medium was replaced with fresh medium 4 hours after transfection. The virus containing medium was collected 48 hours later and filtered through a 0.48 μm filter then mixed with 1/2 volume of concentration medium containing 25.5% PEG 6000 (MilliporeSigma), 0.9 M NaCl, 2.5 mM Na_2_HPO_4_, and 0.4 mM KH_2_PO_4_. The samples were stored at 4ºC overnight then centrifuged at 17,000 g for 1 hour at 4ºC. The resulting pellet containing lentivirus was resuspended with DMEM medium and stored at -80ºC[29].

### 2.12 *In vitro* binding assay

To assess the binding of UBE4A with apoA-I, flag-tagged apoA-I and GST-fused UBE4A were produced in HEK293 cells. Cell lysates were pre-cleaned with protein A/G beads (Santa Cruz Biotechnology), anti-flag-tag antibody was added to cell lysates expressing flag-apoA-I overnight. Flag-apoA-I was pulled down by protein A/G beads and washed three times. Separately, Glutathione Sepharose (GE Life Sciences) was used to pull down GST-fused UBE4A. GST-UBE4A was released by reduced glutathione (50 mM) in a buffer containing 200 mM NaCl, 50 mM Tris (PH 9.0) and protease inhibitors. Purified UBE4A was added to protein A/G agarose bound flag-tagged apoA-I in the presence of InsP_6_, 5PP-InsP_5_, 5-PCP-InsP_5_ (5PCP), 5-PCF_2_Am-InsP_5_ (CF2), 1PP-InsP_5_, 3PP-InsP_5_, InsP_3_, InsP_4_, or InsP_5_ overnight at 4°C. Beads were then collected and washed three times. The samples were loaded with 1.5× SDS loading buffer containing 5% β-mercaptoethanol and boiled for 5 min.

To assess the binding of apoA-I to 5-PCP-InsP_5_, the control resins and 5-PCP-InsP_5_ resins (5PCP resins) were equilibrated with cell lysis buffer. ApoA-I was prepared by using Glutathione Sepharose to pull down GST-fused apoA-I, and the GST was cut out by using PreScission Protease. Purified apoA-I was added onto the control resins and 5PCP resins and incubated overnight at 4°C. The resins were collected and washed three times. The samples were mixed with 1.5× SDS loading buffer containing 5% β-mercaptoethanol and boiled for 5 min. The binding of UBE4A to 5-PCP-InsP_5_ was performed by the same method.

D-*myo*-inositol 1,4,5-trisphosphate (InsP_3_, cat#10008205), D-*myo*-inositol 1,3,4,5-tetrakisphosphate (InsP_4_, cat#60980) and *myo*-inositol 1,3,4,5,6-pentakisphosphate (InsP_5_, cat# 10007784) were purchased from Cayman Chemical. *Myo*-inositol 1,2,3,4,5,6-hexakisphosphate (InsP_6_, cat#P8810) was purchased from MilliporeSigma.

### 2.13 Chemical synthesis

5-PCF_2_Am-InsP_5_ (CF2) and 5-PCP-InsP_5_ (5PCP) were synthesized as previously described[30, 31]. The inositol pyrophosphates 5PP-InsP_5_ was synthesized as described[32], and 1PP-InsP_5_ and 3PP-InsP_5_ were synthesized using similar methods. All synthetic compounds were purified by ion-exchange chromatography, fully characterized by ^1^H, ^31^P and ^13^C NMR spectroscopy and where feasible quantified by total phosphate analysis. 1/3-position 5-PCP-InsP_5_ resin was synthesized as previously described[13]. SC-919 was synthesized as previously described[18].

### 2.14 Immunofluorescence staining

Cultured cells were washed with PBS and then fixed with 4% (w/v) paraformaldehyde for 10 min to preserve morphology while maintaining antigenicity. The samples were washed three times with PBS, and blocked with 10% (v/v) goat serum for 15 min at room temperature. The samples were incubated with primary antibodies (1:200 dilution in 1% BSA) at 4°C overnight. After washing three times with PBS at room temperature, the samples were incubated with Alexa Fluor-conjugated secondary antibodies (1:200 dilution in 1% BSA) for 1 hour at room temperature. The nuclei were stained with DAPI. Pictures were captured by using confocal laser scanning microscopy (Zeiss LSM 800).

### 2.15 Isolation of HDL

The HDL was isolated by using an HDL purification kit (STA-607; Cell Biolabs), following the manufacturer’s protocol. Briefly, 1.25 μL Dextran Solution and 12.5 μL Precipitation Solution were added to 250 μL plasma and incubated on ice for 5 min. The samples were centrifuged at 6000 g for 10 min at 4ºC. The supernatants (200 μL) were mixed with Dextran Solution (12 μL) and Precipitation Solution (30 μL) and incubated for 2 hours at room temperature. The samples were centrifuged at 18,000 g for 30 min at 4ºC. The supernatants were discarded and the pellets were resuspended with HDL Resuspension Buffer (100 μL). The samples were centrifuged at 6000 g for 10 min at 4ºC. The supernatants were discarded and the pellets were resuspended with HDL Wash Solution (120 μL) and mixed for 30 min at 4ºC. The samples were centrifuged at 6000 g for 10 min at 4ºC. The supernatants were collected and mixed with Dextran Removal Solution (18 μL) and incubate for 1 hour at 4ºC. The samples were centrifuged at 6000 g for 10 min at 4ºC. The supernatants were HDL.

### 2.16 Measurement of HDL_2_/HDL_3_

Animals were euthanized by using CO_2_, blood was collected via cardiac puncture, heparin was used as anti-coagulant. The plasma was separated by centrifugation. One volume of 45% (v/v) PEG6000 solution was added to five volumes of plasma and mixed thoroughly. The samples were centrifuged at 1000 g for 15 min to remove LDL and VLDL. The supernatants containing HDL-C were collected. Two volumes of 30% (v/v) PEG6000 was added to three volumes of supernatant and mixed thoroughly. The samples were then centrifuged at 1000 g for 15 min to remove HDL_2_. The supernatants to containing HDL_3_ were collected. HDL-C and HDL_3_-C were determined by using High Density Lipoprotein Cholesterol kit (BioSino Bio-Technology and Science, Beijing, China). The amount of HDL_2_-C was determined by subtraction of HDL_3_-C from HDL-C.

### 2.17 Statistical analysis

Image J was used to analyze western blots. GraphPad Prism 8.0 was used for all statistical analyses. Analyses were conducted in a blinded fashion, and no outliers were excluded. All data were tested for normality using the Shapiro-Wilk normality test. For normally distributed data, comparisons between two groups were performed using the unpaired Student’s *t*-test, and comparisons among more than two groups were performed using One-way analysis of variance (ANOVA), corrected for multiple comparisons using the Bonferroni post-hoc test. For non-normally distributed data, Mann-Whitney U test was performed. All statistical data were presented as mean±SEM. Differences were considered significant at *P < 0.05, **P < 0.01, and ***P < 0.001.

## 3 Results

### 3.1 Deleting IP6K1 elevates circulating apoA-I levels

**Fig. 1:**
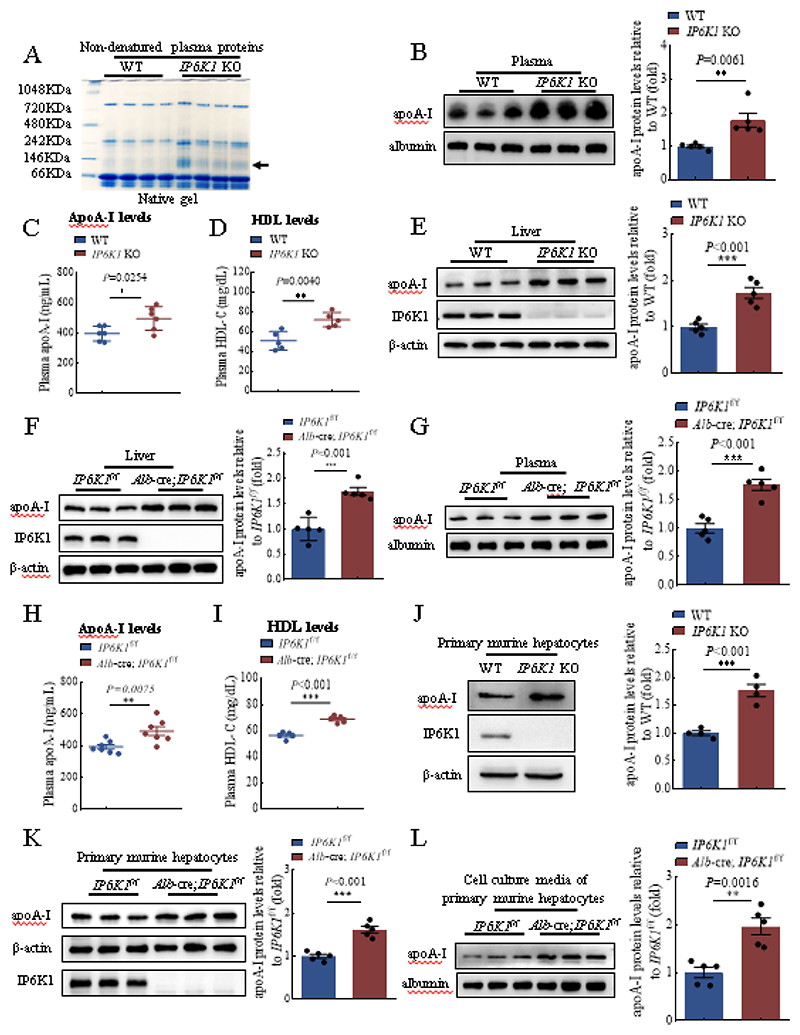
Knockout of IP6K1 boosted the protein levels of apoA-I in the liver, resulting in an increase in HDL in the plasma. **A**, Non-denatured plasma proteins of WT and *IP6K1* KO mice were separated by protein gel electrophoresis in a native gel, which was stained by Coomassie blue. The protein band (arrow) was identified by mass spectrometry as apoA-I. n=4 mice per group. **B**, Protein levels of apoA-I in the plasma of WT and *IP6K1* KO mice. Data represent mean±SEM, Student’s *t*-test, n=5 mice per group. **C**, ApoA-I concentrations in the plasma of WT and *IP6K1* KO mice were determined by ELISA assay. Data were presented as mean ± SEM, Student’s t-test, n = 6 mice per group. **D**, Levels of HDL cholesterol in the plasma of WT and *IP6K1* KO mice. Data represent mean±SEM, Student’s *t*-test, n=5 mice per group. **E**, Protein levels of apoA-I in the livers of WT and *IP6K1* KO mice. Data represent mean±SEM, Student’s *t*-test, n=5 mice per group. **F**, Protein levels of apoA-I in the livers of control (*IP6K1*^f/f^) and hepatocyte-specific *IP6K1* KO mice (*Alb*-cre; *IP6K1*^f/f^). Data represent mean±SEM, Student’s *t*-test, n=5 mice per group. **G**, Protein levels of apoA-I in the plasma of *IP6K1*^f/f^ and *Alb*-cre; *IP6K1*^f/f^ mice. Data represent mean±SEM, Student’s *t*-test, n=5 mice per group. **H**, ApoA-I concentrations in the plasma of *IP6K1*^f/f^ and *Alb*-cre; *IP6K1*^f/f^ mice were determined by ELISA assay. Data were presented as mean ± SEM, Student’s t-test, n=7 mice per group. **I**, Levels of HDL cholesterol in the plasma of *IP6K1*^f/f^ and *Alb*-cre; *IP6K1*^f/f^ mice. Data represent mean±SEM, Student’s *t*-test, n=5 mice per group. **J**, Protein levels of apoA-I in the *in vitro* cultured primary murine hepatocytes of WT and *IP6K1* KO mice. Data represent mean±SEM, Student’s *t*-test, n=4 independent repeats. **K**, Protein levels of apoA-I in the *in vitro* cultured primary murine hepatocytes of the *IP6K1*^f/f^ and *Alb*-cre; *IP6K1*^f/f^ mice. Data represent mean±SEM, Student’s *t*-test, n=5 independent repeats. **L**, Protein levels of apoA-I in the cell culture media of primary murine hepatocytes of the *IP6K1*^f/f^ and *Alb*-cre; *IP6K1*^f/f^ mice. Data represent mean±SEM, Student’s *t*-test, n=5 independent repeats.

We collected the plasma of *IP6K1* knockout (KO) mice and their wild-type (WT) littermates. The plasma proteins were separated in their non-denatured state using native gel and visualized with Coomassie blue staining. Results showed that a protein band, which was identified by mass spectrometry as apoA-I, appeared noticeably darker in the *IP6K1* KO preparations (Fig. 1A). This suggested that a higher protein level of apoA-I was present in the *IP6K1* KO plasma, which was confirmed by western blots (Fig. 1B). The increased apoA-I was further validated by ELISA assay (Fig. 1C). ApoA-I is the major structural and functional protein of HDL, we thus examined the plasma levels of HDL, which were also higher in the *IP6K1* KOs than in WTs (Fig. 1D). In contrast, the plasma levels of LDL were lower in the *IP6K1* KOs, although not significantly so (Suppl. Fig. 1A). The plasma levels of total cholesterol were similar between WTs and *IP6K1* KOs (Suppl. Fig. 1B).

### 3.2 Deleting IP6K1 increases apoA-I protein levels in hepatocytes

Hepatocytes are the major source of apoA-I and produce more than 80% of circulating HDL[33], besides small intestine also expresses apoA-I[34]. Deletion of IP6K1 upregulates apoA-I in the liver (Fig. 1E), however, does not upregulate apoA-I in the small intestine (Suppl. Fig. 2). To validate the regulation of apoA-I by IP6K1, we generated hepatocyte-specific *IP6K1* KO mice (*Alb*-cre; *IP6K1*^f/f^). Consistently, the apoA-I protein levels were higher in the livers of hepatocyte-specific *IP6K1* KO animals than in the controls (Fig. 1F). The hepatocyte-specific *IP6K1* KO mice also displayed higher plasma levels of apoA-I and HDL (Fig. 1G-I). The plasma levels of LDL cholesterol and total cholesterol were not significantly different between hepatocyte-specific *IP6K1* KO mice and control mice (Suppl. Fig. 3A, B).

We compared the apoA-I protein levels in the primary murine hepatocytes from the global *IP6K1* KO mice and their littermate controls. Results showed that the protein levels of apoA-I were higher in the *IP6K1* KO preparations (Fig. 1J). Similarly, the primary murine hepatocytes from the hepatocyte-specific *IP6K1* KO mice also displayed higher expression levels of apoA-I (Fig. 1K). Furthermore, the protein levels of apoA-I were higher in the cell culture media of the hepatocyte-specific *IP6K1* KO primary murine hepatocytes than in controls (Fig. 1L and Suppl. Fig. 4). These results confirmed that deletion of IP6K1 upregulates apoA-I in hepatocytes.

### 3.2 Deleting IP6K1 does not affect the protein levels of apoA-V, apoB, or apoE

We asked whether deletion of IP6K1 in hepatocytes affects the protein components of HDL. To answer that question, we collected the plasma of hepatocyte-specific *IP6K1* KO and control mice and purified the HDL. The HDL proteins were separated by SDS PAGE and visualized with Coomassie blue staining. There were no observable differences between the two groups, indicating that deletion of IP6K1 does not affect the protein components of HDL (Suppl. Fig. 5).

We asked whether deletion of IP6K1 affects other apolipoproteins. To answer that question, we run western blots to examine the plasma levels of apoA-V, apoB, and apoE in the whole-body *IP6K1* KO mice and their WT littermates. Results show that their protein levels were similar between WT and *IP6K1* KO preparations, indicating that IP6K1 does not regulate their expression levels (Suppl. Fig. 6).

### 3.4 Pharmacological inhibition of IP6K1 increases apoA-I protein levels *in vivo*

**Fig. 2:**
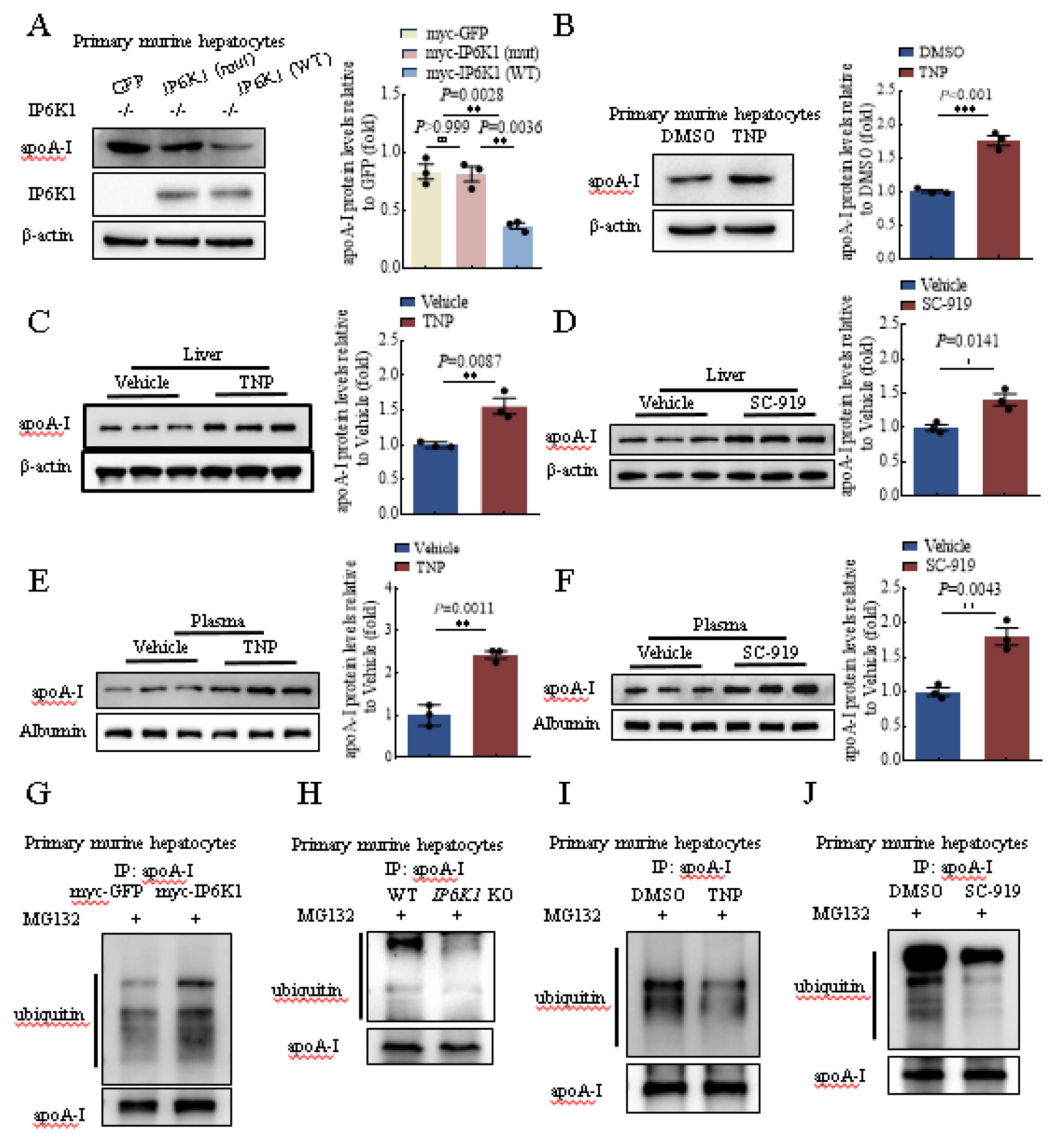
Pharmacological inhibition of IP6K increased apoA-I protein levels. **A**, Overexpressing WT but not mutant IP6K1 in the *IP6K1* KO murine hepatocytes rescued apoA-I protein levels. Data represent mean±SEM, One-way ANOVA, n=3 independent repeats. **B**, Inhibiting IP6K activity by TNP (3 μM, 24 hours) treatment upregulated apoA-I in primary murine hepatocytes. Data represent mean±SEM, Student’s *t*-test, n=3 independent repeats. **C**, WT mice were treated with TNP (20 mg/kg) for 1 week. The apoA-I protein levels in the livers were upregulated. Data represent mean±SEM, Student’s *t*-test, n=3 mice per group. **D**, WT mice were treated with SC-919 (10 mg/kg) for 1 week. The apoA-I protein levels in the livers were upregulated. Data represent mean±SEM, Student’s *t*-test, n=3 mice per group. **E**, Administration of TNP (20 mg/kg, 1 week) increased apoA-I protein levels in the plasma. Data represent mean±SEM, Student’s *t*-test, n=3 mice per group. **F**, Administration of SC-919 (10 mg/kg, 1 week) increased apoA-I protein levels in the plasma. Data represent mean±SEM, Student’s *t*-test, n=3 mice per group. **G**, Myc-IP6K1 was overexpressed in primary murine hepatocytes. Myc-GFP was overexpressed as a control. The cells were treated with MG132 (10 μM, 4 hours) to block proteasomal activity. ApoA-I was immunoprecipitated and blotted for ubiquitin. **H**, WT and *IP6K1* KO primary murine hepatocytes were treated with MG132 (10 μM, 4 hours) to block proteasomal activity. ApoA-I was immunoprecipitated and blotted for ubiquitin. **I**, Primary murine hepatocytes were treated with TNP (3 μM, 24 hours) or DMSO. The cells were then treated with MG132 (10 μM, 4 hours) to block proteasomal activity. ApoA-I was immunoprecipitated and blotted for ubiquitin. **J**, Primary murine hepatocytes were treated with SC-919 (1 μM, 24 hours) or DMSO. The cells were then treated with MG132 (10 μM, 4 hours) to block proteasomal activity. ApoA-I was immunoprecipitated and blotted for ubiquitin.

IP6K1 can function in a kinase-independent manner [35]. To determine whether the kinase activity of IP6K1 was required for reducing apoA-I expression, we rescued IP6K1 protein expression in *IP6K1* KO murine hepatocytes by overexpressing WT IP6K1 or a kinase activity defective mutant IP6K1. Western blots showed that WT IP6K1, but not the mutant IP6K1, lowered the apoA-I protein levels (Fig. 2A), indicating that the kinase activity of IP6K1 is required for reducing apoA-I. In support of this, blocking IP6K enzymatic activity by TNP treatment elevated apoA-I in primary murine hepatocytes (Fig. 2B), as well as in the cell culture media (Suppl. Fig. 7).

To investigate whether pharmacological inhibition of IP6K1 could increase apoA-I expression *in vivo*, we administered TNP and SC-919, a newly developed IP6K inhibitor[18], to animals. Both TNP and SC-919 treatment increased apoA-I protein levels in the livers (Fig, 2C, D), and in the plasma (Fig. 2E, F). The pharmacological inhibition of IP6K1-induced elevation of apoA-I was further validated by ELISA assay (Suppl. Fig. 8).

### 3.5 Diphosphoinositol pentakisphosphate kinases (PPIP5Ks) do not regulate apoA-I

PPIP5Ks produce 1PP-InsP_5_, a regio-isomer of 5PP-InsP_5_, which is the major product of IP6K1. Besides, PPIP5Ks can also utilize 5PP-InsP_5_, to generate InsP_8_[36]. To test whether PPIP5Ks also regulate apoA-I protein levels, we deleted PPIP5K1 and PPIP5K2 in murine hepatocyte AML12 cells by shRNA transduction. Results showed that neither deletion of PPIP5K1 nor PPIP5K2 affected the protein levels of apoA-I, indicating that PPIP5Ks may not play a major role in regulating apoA-I protein levels (Suppl. Fig. 9).

### 3.6 IP6K1 does not affect the protein levels of ABCA1 or ABCG1

The ATP binding cassette subfamily A member 1 (ABCA1) plays a critical role in the biogenesis of nascent HDL[33]. It has been shown that newly synthesized apoA-I interacts with ABCA1 intracellularly and as it translocates to the plasma membrane to promote efflux[37, 38]. We examined the protein levels of ABCA1 and ABCG1 in the primary hepatocytes of hepatocyte-specific *IP6K1* KO and control animals. Results showed that their protein levels were similar between hepatocyte-specific *IP6K1* KO and control preparations, indicating that IP6K1 does not affect the expression levels of ABCA1 or ABCG1 (Suppl. Fig. 10A). To test whether deletion of IP6K1 affects the binding of apoA-I with ABCA1, we performed immunoprecipitations of apoA-I in WT and *IP6K1* KO primary murine hepatocytes. Results showed that equal amounts of ABCA1 were co-pulled down by apoA-I in WT and *IP6K1* KO primary murine hepatocytes, indicating that deletion of IP6K1 does not affect the interactions of apoA-I with ABCA1 (Suppl. Fig. 10B).

### 3.7 Blocking IP6K1 activity elevates apoA-I protein levels by enhancing its stability

IP6K1 does not regulate apoA-I at the transcriptional level, because the mRNA levels of apoA-I in primary hepatocytes were similar between WT and *IP6K1* KO preparations (Suppl. Fig. 11A). Similarly, hepatocyte-specific deletion of IP6K1 did not affect apoA-I mRNA levels (Suppl. Fig. 11B).

It has been reported that deletion of IP6K1 reduces protein O-GlcNAcylation levels in the liver[39]. To test whether O-GlcNAcylation plays a role in the regulation of apoA-I by IP6K1, we immunoprecipitated apoA-I in the primary hepatocytes of WT and *IP6K1* KOs and blotted O-GlcNAc. Results showed that the O-GlcNAcylation levels were similar between WT and *IP6K1* KO preparations (Suppl. Fig. 12), indicating O-GlcNAcylation is not involved.

We previously showed that IP6K1 can affect protein stability [19, 40]. To test whether IP6K1 decreased apoA-I protein levels by inducing its protein degradation, we performed immunoprecipitations and western blots to determine the ubiquitination levels of apoA-I, a marker for degradation. Results showed that overexpressing IP6K1 in the *in vitro* cultured primary murine hepatocytes increased the ubiquitination levels of apoA-I (Fig. 2G), whereas deletion of IP6K1 reduced the ubiquitination levels of apoA-I in the primary murine hepatocytes (Fig. 2H).

To test whether pharmacological inhibition of IP6K could lower the ubiquitination levels of apoA-I, we treated the *in vitro* cultured primary murine hepatocytes with TNP or SC-919. Results showed that both TNP and SC-919 treatment reduced apoA-I ubiquitination levels (Fig. 2I, J). Thus, the above results demonstrated that blocking IP6K1 activity elevates apoA-I protein levels by enhancing its stability.

### 3.8 IP6K1 formed a complex with apoA-I and UBE4A

**Fig. 3:**
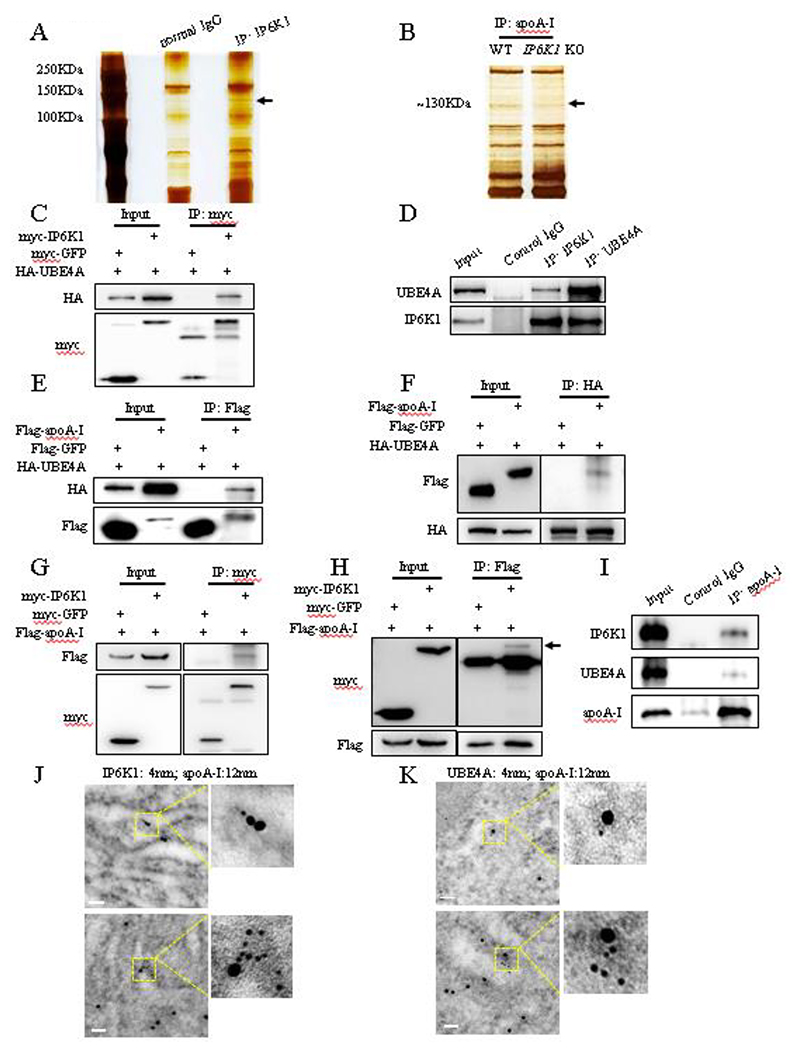
IP6K1 formed a complex with apoA-I and UBE4A. **A**, IP6K1 was immunoprecipitated in primary murine hepatocytes. Protein electrophoresis and silver staining were performed to look for its binding partners. The protein band (arrow) was identified by mass spectrometry as UBE4A. **B**, ApoA-I was immunoprecipitated in hepatocytes of WT and *IP6K1* KOs. Protein electrophoresis and silver staining were performed to look for its binding partners. One protein band with molecular weight ~130 KDa (arrow) appeared darker in the WT preparations. **C**, Myc-IP6K1 and HA-UBE4A were overexpressed together, myc-GFP was overexpressed as a control. Immunoprecipitation of myc-IP6K1 co-pulled down HA-UBE4A. **D**, Immunoprecipitations of endogenous IP6K1 and UBE4A in primary murine hepatocytes co-pulled down each other. **E and F**, Flag-apoA-I and HA-UBE4A were overexpressed together, flag-GFP was overexpressed as a control. (E) Immunoprecipitation of flag-apoA-I co-pulled down HA-UBE4A. (F) Immunoprecipitation of HA-UBE4A co-pulled down flag-apoA-I. **G and H**, Myc-IP6K1 and flag-apoA-I were overexpressed together, myc-GFP was overexpressed as a control. (G) Immunoprecipitation of myc-IP6K1 co-pulled down flag-apoA-I. (H) Immunoprecipitation of flag-apoA-I co-pulled down myc-IP6K1 (arrow). **I**, Endogenous IP6K1 and UBE4A were co-pulled down by endogenous apoA-I in primary murine hepatocytes. **J**, IP6K1 (4 nm) and apoA-I (12 nm) were labeled in murine hepatocytes by immuno-electron microscopy. Scale bar 50 nm. **K**, UBE4A (4 nm) and apoA-I (12 nm) were labeled in murine hepatocytes by immuno-electron microscopy. Scale bar 50 nm.

To dissect the mechanism by which IP6K1 induces apoA-I degradation, we performed immunoprecipitations of IP6K1 and apoA-I to seek their binding partners. By employing protein electrophoresis, silver staining, and mass spectrometry, we identified that UBE4A, an E3 ubiquitin ligase, was co-pulled down by IP6K1 (Fig. 3A, C, and D). Intriguingly, we found that UBE4A could also be co-immunoprecipitated by apoA-I (Fig. 3B, E, and F).

We first validated the binding of IP6K1 to UBE4A. HA-tagged UBE4A and myc-tagged IP6K1 were overexpressed together in HEK 293 cells. Immunoprecipitation of myc-IP6K1 co-pulled down HA-UBE4A (Fig. 3C). We then examined the binding of endogenous IP6K1 and UBE4A. Immunoprecipitations of endogenous IP6K1 and UBE4A co-pulled down each other in primary murine hepatocytes (Fig. 3D). We next confirmed the interaction of apoA-I with UBE4A by performing co-immunoprecipitations of HA-tagged UBE4A and flag-tagged apoA-I (Fig. 3E, F).

We asked if IP6K1 forms a complex with apoA-I and UBE4A. To test this possibility, we examined whether IP6K1 interacts with apoA-I. Myc-tagged IP6K1 and flag-tagged apoA-I were overexpressed together in HEK 293 cells. Immunoprecipitation of myc-IP6K1 by utilizing anti-myc antibodies co-pulled down flag-apoA-I (Fig. 3G). Immunoprecipitation of flag-apoA-I by utilizing anti-flag antibodies co-pulled down myc-IP6K1 (Fig. 3H). We further confirm that endogenous IP6K1 and UBE4A can be co-immunoprecipitated by endogenous apoA-I in primary murine hepatocytes (Fig. 3I), indicating that IP6K1 forms a complex with apoA-I and UBE4A.

ApoA-I is synthesized and secreted in the endoplasmic reticulum/Golgi apparatus pathway. We performed subcellular fractionations and western blots to show that IP6K1 was present in the endoplasmic reticulum of hepatocytes (Suppl. Fig. 13). The colocalization of IP6K1 and apoA-I (Fig. 3J), and the colocalization of UBE4A and apoA-I (Fig. 3K) in hepatocytes were observed by utilizing immuno-electron microscopy.

### 3.9 UBE4A mediates apoA-I ubiquitination and degradation

**Fig. 4:**
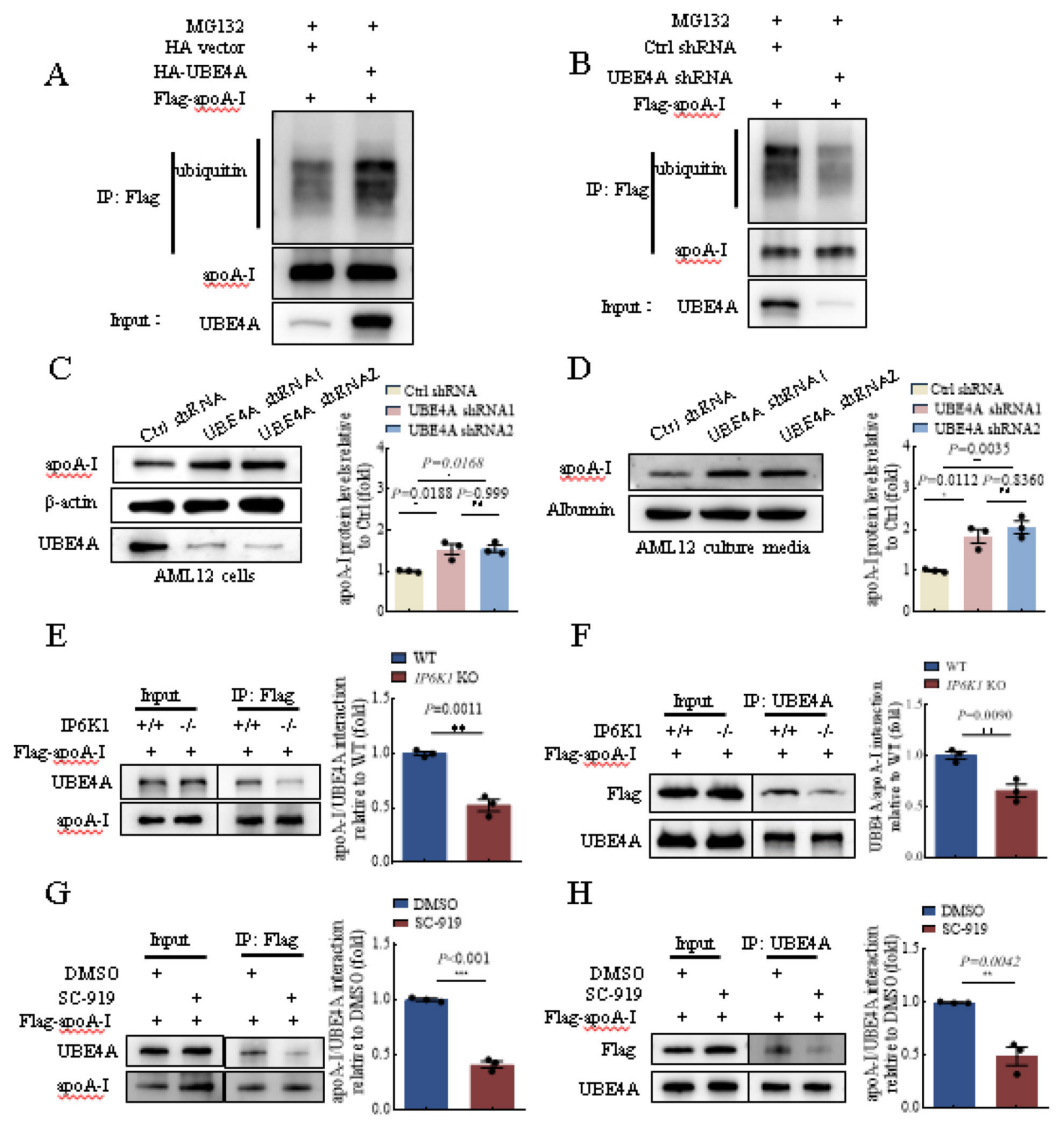
UBE4A was recruited by IP6K1 to ubiquitinate apoA-I. **A**, Flag-apoA-I and HA-UBE4A were overexpressed together in primary murine hepatocytes. The cells were treated with M132 (10 μM, 4 hours) to block proteasomal activity. Flag-apoA-I was immunoprecipitated and blotted for ubiquitin. **B**, Flag-apoA-I was overexpressed in primary murine hepatocytes, and UBE4A was deleted by shRNA transduction (the shRNA2 as in panel D). The cells were treated with M132 (10 μM, 4 hours) to block proteasomal activity. Flag-apoA-I was immunoprecipitated and blotted for ubiquitin. **C and D**, UBE4A was deleted in AML12 murine hepatocytes. (C) Deletion of UBE4A upregulated apoA-I protein levels. Data represent mean±SEM, One-way ANOVA, n=3 independent repeats. (D) The protein levels of apoA-I in the cell culture media were higher in the UBE4A-deleted preparations. Data represent mean±SEM, One-way ANOVA, n=3 independent repeats. **E and F**, Flag-apoA-I was overexpressed in WT and *IP6K1* KO primary murine hepatocytes. (E), Immunoprecipitation of flag-apoA-I co-pulled down less UBE4A in the *IP6K1* KOs. Data represent mean±SEM, Student’s *t*-test, n=3 independent repeats. (F), Immunoprecipitation of UBE4A co-pulled down less flag-apoA-I in the *IP6K1* KOs. Data represent mean±SEM, Student’s *t*-test, n=3 independent repeats. **G and H**, Flag-apoA-I was overexpressed in primary murine hepatocytes. The cells were treated with IP6K inhibitor SC-919 (1 μM, 24 hours). (G), Immunoprecipitation of flag-apoA-I co-pulled down less UBE4A in the SC-919-treated cells. Data represent mean±SEM, Student’s *t*-test, n=3 independent repeats. (H), Immunoprecipitation of UBE4A co-pulled down less flag-apoA-I in the SC-919-treated cells. Data represent mean±SEM, Student’s *t*-test, n=3 independent repeats.

We examined whether UBE4A could induce apoA-I ubiquitination and degradation in hepatocytes. Overexpressing UBE4A in primary murine hepatocytes raised the ubiquitination levels of apoA-I (Fig. 4A), whereas, deleting UBE4A by shRNA transduction in primary murine hepatocytes decreased the ubiquitination levels of apoA-I (Fig. 4B). We utilized the murine hepatocyte AML12 cells to confirm the regulation of apoA-I by UBE4A. Results showed that deleting UBE4A increased the protein levels of apoA-I in AML12 cells (Fig. 4C), and also elevated the protein levels of apoA-I in the cell culture media (Fig. 4D).

### 3.10 UBE4A is recruited by IP6K1 to ubiquitinate apoA-I

To test the possibility that IP6K1 affects the binding of UBE4A to apoA-I, we examined the interactions of UBE4A with apoA-I in WT and *IP6K1* KO murine hepatocytes. Flag-tagged apoA-I was overexpressed in WT and *IP6K1* KO murine hepatocytes. Less UBE4A was co-immunoprecipitated by flag-apoA-I in the *IP6K1* KO cells than in WTs (Fig. 4E). Similarly, lower amounts of flag-apoA-I were co-pulled down by UBE4A in the *IP6K1* KO hepatocytes than in WTs (Fig. 4F), indicating that deletion of IP6K1 reduced the binding of UBE4A to apoA-I.

To study the impact of 5PP-InsP_5_, the primary product of IP6K1 on the binding of UBE4A to apoA-I, we utilized SC-919, a newly developed IP6K inhibitor, to block 5PP-InsP_5_ biosynthesis in the *in vitro* cultured primary murine hepatocytes. Flag-tagged apoA-I was overexpressed in the primary murine hepatocytes to examine the binding of UBE4A to apoA-I. Less UBE4A was co-immunoprecipitated by flag-apoA-I in the SC-919-treated cells (Fig. 4G). Similarly, lower amounts of flag-apoA-I were co-pulled down by UBE4A in the SC-919-treated cells (Fig. 4H), indicating that 5PP-InsP_5_ mediated the interaction of UBE4A with apoA-I.

### 3.11 IP6K1 generates 5PP-InsP_5_ to enhance the binding of UBE4A to apoA-I

**Fig. 5:**
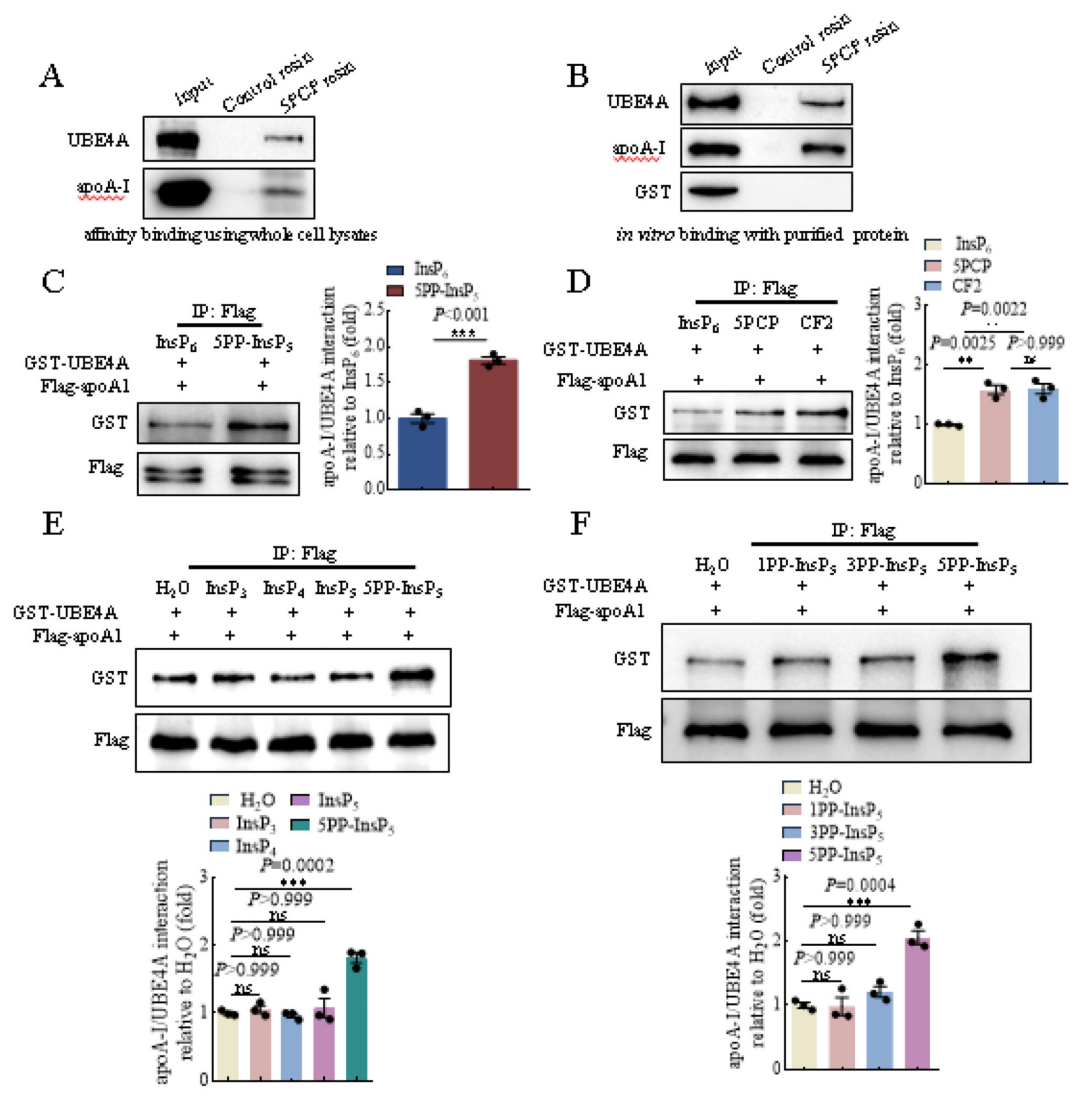
5PP-InsP_5_ enhanced the binding of UBE4A to apoA-I. **A**, 5PCP resins pulled down endogenous UBE4A and apoA-I in whole cell lysates. **B**, 5PCP resins pulled down purified UBE4A and apoA-I, but not GST in an *in vitro* protein binding assay. **C**, 5PP-InsP_5_ enhanced the binding of UBE4A to apoA-I. Data represent mean±SEM, Student’s *t*-test, n=3 independent repeats. **D**, Both 5-PCP-InsP_5_ (5PCP) and 5-PCF_2_Am-InsP_5_ (CF2) promoted the binding of UBE4A to apoA-I. Data represent mean±SEM, One-way ANOVA, n=3 independent repeats. **E**, 5PP-InsP_5_ but not InsP_3_, InsP_4_ or InsP_5_ enhanced the binding of UBE4A to apoA-I. Data represent mean±SEM, One-way ANOVA, n=3 independent repeats. **F**, 5PP-InsP_5_ but not 1PP-InsP_5_ or 3PP-InsP_5_ enhanced the binding of UBE4A to apoA-I. Data represent mean±SEM, One-way ANOVA, n=3 independent repeats.

To study the mechanism by which 5PP-InsP_5_ strengthened the binding of UBE4A to apoA-I, we examined the possibility that 5PP-InsP_5_ binds UBE4A and/or apoA-I. We utilized resin bound 5PCP-InsP_5_ (5PCP), a non-hydrolyzable analog of 5PP-InsP_5_, as a bait. The endogenous UBE4A and apoA-I were pulled down by 5PCP beads in whole cell lysates (Fig. 5A). 5PCP beads also pulled down purified UBE4A and apoA-I but not GST in the *in vitro* binding assay (Fig. 5B), suggesting that 5PP-InsP_5_ binds UBE4A and apoA-I.

To study whether 5PP-InsP_5_ could directly mediate the binding of UBE4A to apoA-I, we performed *in vitro* binding assays by utilizing purified proteins. Compared with InsP_6_, the immediate precursor of 5PP-InsP_5_, 5PP-InsP_5_ augmented the binding of UBE4A to apoA-I (Fig. 5C). 5-PCP-InsP_5_ (5PCP) and 5-PCF_2_Am-InsP_5_ (CF2) are non-hydrolyzable analogs of 5PP-InsP_5_, possessing similar structural and biochemical properties, except that 5PCP and CF2 are not able to transfer their β-phosphate[30, 41]. Both 5PCP and CF2 strengthened the interaction of UBE4A with apoA-I in the *in vitro* binding assays (Fig. 5D).

We compared 5PP-InsP_5_ with the lower inositol phosphates Ins(1,4,5)P_3_, Ins(1,3,4,5)P_4_ and Ins(1,3,4,5,6)P_5_ in mediating the binding of UBE4A to apoA-I in the *in vitro* binding assays. 5PP-InsP_5_ but not Ins(1,4,5)P_3_, Ins(1,3,4,5)P_4_ or Ins(1,3,4,5,6)P_5_ enhanced the interaction of UBE4A with apoA-I (Fig. 5E).

1-Diphosphoinositol pentakisphosphate (1PP-InsP_5_) and 3-diphosphoinositol pentakisphosphate (3PP-InsP_5_) are regio-isomers of 5PP-InsP_5_. The pyrophosphate group of 1PP-InsP_5_, 3PP-InsP_5_, and 5PP-InsP_5_ is at the 1-, 3-, and 5-position of the inositol ring, respectively. The *in vitro* binding assay demonstrated that 5PP-InsP_5_ but not 1PP-InsP_5_ or 3PP-InsP_5_ enhanced the binding of UBE4A to apoA-I (Fig. 5F).

These above results demonstrated that 5PP-InsP_5_ binds UBE4A and apoA-I, and 5PP-InsP_5_ enhanced the binding of UBE4A to apoA-I, directly.

### 3.12 Deletion of IP6K1 in hepatocytes does not affect the body weight, liver weight, blood glucose, and blood triglyceride levels

IP6K1 has been proposed to be a potential therapeutic target for certain diseases treatment[15, 17, 42]. To test whether deletion of IP6K1 in hepatocytes affects the body weight, liver weight, blood glucose, and blood triglyceride levels, we examined those parameters in hepatocyte-specific *IP6K1* KO and control mice. Results showed that the body weights, liver weights, blood glucose levels and blood triglyceride levels at baseline were similar between hepatocyte-specific *IP6K1* KO and control mice (Suppl. Fig. 14A), which is consistent with previous report[39].

### 3.13 Hepatocyte-specific deletion of IP6K1 elevates circulating apoA-I, augments cholesterol efflux, and attenuates atherosclerosis

Higher plasma levels of apoA-I are commonly associated with a higher ability of reverse cholesterol transport and lower rates of atherosclerosis[43]. We studied the physiological relevance of the elevated apoA-I in hepatocyte-specific *IP6K1* KO mice. These animals were injected with recombinant adeno-associated virus encoding a gain-of-function mutant PCSK9 (AAV-PCSK9) and fed with a Western diet to induce atherosclerosis. We examined the expression levels of PCSK9 and its downstream target LDL receptor (LDLR) in the livers to confirm the effectiveness of AAV-PCSK9 (Suppl. Fig. 15). The levels of plasma LDL increased from basal levels of ~22 mg/dL to ~300 mg/dL and the levels of total cholesterol increased from basal levels of ~100 mg/dL to ~1000 mg/dL after treatment with AAV-PCSK9 and Western diet (Suppl. Fig. 16). The body weights, liver weights, blood glucose levels and blood triglyceride levels were similar between hepatocyte-specific *IP6K1* KO and control mice after treatment with AAV-PCSK9 and Western diet (Suppl. Fig. 14B).

**Fig. 6:**
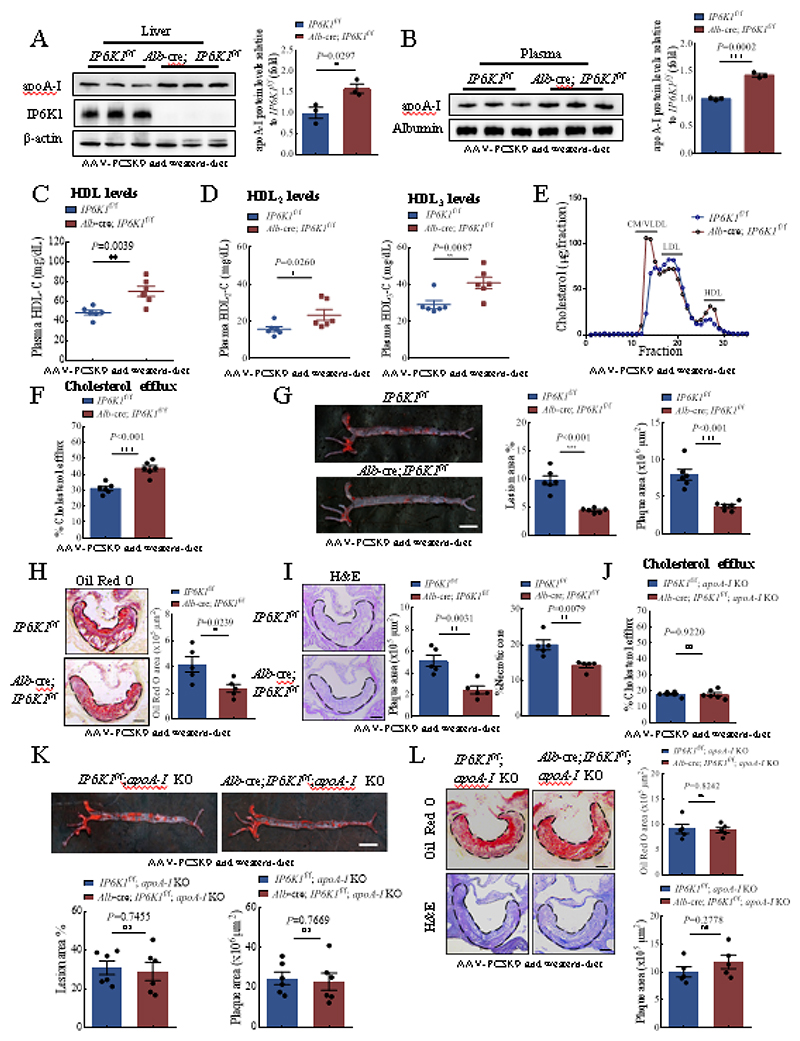
Knockout of IP6K1 augmented cholesterol efflux and attenuated atherosclerosis. **A-I**, Hepatocyte-specific *IP6K1* KO (*Alb*-cre; *IP6K1*^f/f^) and control (*IP6K1*^f/f^) mice were injected with AAV-PCSK9 and fed with a Western diet. (A), The protein levels of apoA-I were higher in the livers of *Alb*-cre; *IP6K1*^f/f^ mice than in controls (*IP6K1*^f/f^). Data represent mean±SEM, Student’s *t*-test, n=3 mice per group. (B), The protein levels of apoA-I were higher in the plasma of *Alb*-cre; *IP6K1*^f/f^ mice than in controls (*IP6K1*^f/f^). Data represent mean±SEM, Student’s *t*-test, n=3 mice per group. (C), HDL levels were higher in the plasma of *Alb*-cre; *IP6K1*^f/f^ mice than in controls (*IP6K1*^f/f^). Data represent mean±SEM, Student’s *t*-test, n=6 mice per group. (D), HDL_2_ and HDL_3_ levels were higher in the plasma of *Alb*-cre; *IP6K1*^f/f^ mice than in controls (*IP6K1*^f/f^). Data represent mean±SEM, Mann-Whitney U test, n=6 mice per group. (E), Pooled plasma samples were fractionated by FPLC for cholesterol analysis. VLDL, very-low-density lipoprotein. CM, Chylomicrons. n=6 mice per group. (F), The plasma of *Alb*-cre; *IP6K1*^f/f^ mice and *IP6K1*^f/f^ mice were collected and applied to the cellular cholesterol efflux assay. The *Alb*-cre; *IP6K1*^f/f^ preparations displayed higher cholesterol efflux activities. Data represent mean±SEM, Student’s *t*-test, n=6 mice per group. (G), Oil Red O staining of the whole aorta. Data represent mean±SEM, Student’s *t*-test, n=6 mice per group. Scale bar 5mm. (H), Oil Red O staining of the aorta root. Data represent mean±SEM, Student’s *t*-test, n=5 mice per group. Scale bar 100 μm. (I), H&E staining of the aorta root. Data represent mean±SEM, Student’s *t*-test for plaque area, Mann-Whitney U test for necrotic core, n=5 mice per group. Scale bar 100 μm. **J-L**, The *IP6K1*^f/f^; *apoA-I* KO mice and *Alb*-cre; *IP6K1*^f/f^; *apoA-I* KO mice were injected with AAV-PCSK9 and fed with a Western diet. (J), The plasma of the *IP6K1*^f/f^; *apoA-I* KO mice and the *Alb*-cre; *IP6K1*^f/f^; *apoA-I* KO mice were collected and applied to the cellular cholesterol efflux assay. There were no significant differences between the two groups. Data represent mean±SEM, Student’s *t*-test, n=6 mice per group. (K), Oil Red O staining of the whole aorta. Data represent mean±SEM, Mann-Whitney U test (lesion%), Student’s *t*-test (plaque area), n=6 mice per group. Scale bar 5 mm. (L), H&E and Oil Red O staining of the aorta root. Data represent mean±SEM, Student’s *t*-test, n=5 mice per group. Scale bar 100 μm.

The protein levels of apoA-I were still higher in the livers of hepatocyte-specific *IP6K1* KO mice compared to controls after AAV-PCSK9 and Western diet treatment (Fig. 6A). Similarly, the plasma levels of apoA-I and HDL were still higher in the hepatocyte-specific *IP6K1* KOs than in controls after AAV-PCSK9 and Western diet treatment (Fig. 6B, C). We separated the HDL particles to examine the distribution of the increased apoA-I. HDL_2_ and HDL_3_ are two subclasses of HDL. HDL_3_ is smaller and contains less cholesterol than the larger, cholesterol-rich HDL_2_[44]. Both HDL_2_ and HDL_3_ were higher in the hepatocyte-specific *IP6K1* KO preparations (Fig. 6D). We performed fast protein liquid chromatography to separate the whole plasma of the AAV-PCSK9 and Western diet-treated animals and examined cholesterol levels in the fractions. Results showed that the HDL cholesterol levels were higher while the LDL cholesterol levels were lower in the hepatocyte-specific *IP6K1* KO than in control preparations (Fig. 6E).

We tested the possibility that the elevated plasma levels of apoA-I induced by IP6K1 deletion augments cholesterol efflux. We collected the plasma of the hepatocyte-specific *IP6K1* KO and control animals after treatment with AAV-PCSK9 and Western diet, and performed cellular cholesterol efflux assay. The hepatocyte-specific *IP6K1* KO reparations displayed a higher ability in mediating cholesterol efflux compared to controls (Fig. 6F). To confirm the increased cholesterol efflux is due to increased amounts of apoA-I that will accept cholesterol, we purified apoA-I from the *in vitro* cultured hepatocyte-specific *IP6K1* KO and control hepatocytes. The *in vitro* cholesterol efflux assay demonstrated that equal amounts of apoA-I from the hepatocyte-specific *IP6K1* KO and control hepatocytes mediate similar levels of cholesterol efflux (Suppl. Fig. 17).

We next determined the atherosclerotic lesions in the AAV-PCSK9 and Western diet-treated animals. Oil Red O-staining showed that there were fewer atherosclerotic lesions in the aortas of the hepatocyte-specific *IP6K1* KO mice compared to controls (Fig. 6G). The atherosclerotic plaques in the aortic root were also smaller with reduced necrotic core in the hepatocyte-specific *IP6K1* KO mice than in controls revealed by H&E staining and Oil Red O-staining (Fig. 6H, I).

### 3.14 ApoA-I mediates the IP6K1 deletion-induced atheroprotection

To determine the role of apoA-I in mediating the atheroprotective effects of IP6K1 deletion, we generated mice with both apoA-I deletion and hepatocyte-specific IP6K1 deletion (*Alb*-cre; *IP6K1*^f/f^; *apoA-I* KO), and generated the *IP6K1*^f/f^; *apoA-I* KO mice as controls. These animals were injected with AAV-PCSK9 and fed with a Western diet to induce atherosclerosis. We then collected plasma to measure LDL, HDL and total cholesterol levels and assess cholesterol efflux. The levels of plasma LDL were around 300 mg/dL and total cholesterol were approximately 1100 mg/dL in both the *Alb*-cre; *IP6K1*^f/f^; *apoA-I* KO mice and the *IP6K1*^f/f^; *apoA-I* KO mice after treatment with AAV-PCSK9 and Western diet (Suppl. Fig. 18A, B). Deleting IP6K1 did not increase HDL in the *apoA-I* KO mice (Suppl. Fig. 18C), indicating that knockout of apoA-I negates the IP6K1 deletion-induced upregulation of HDL. Deletion of IP6K1 in the apoA-I null mice failed to augment cholesterol efflux (Fig. 6J), and did not alleviate atherosclerosis (Fig. 6K, L), confirming that the atheroprotective effects of hepatocyte-specific IP6K1 deletion require apoA-I.

## 4 Discussion

**Fig. 7:**
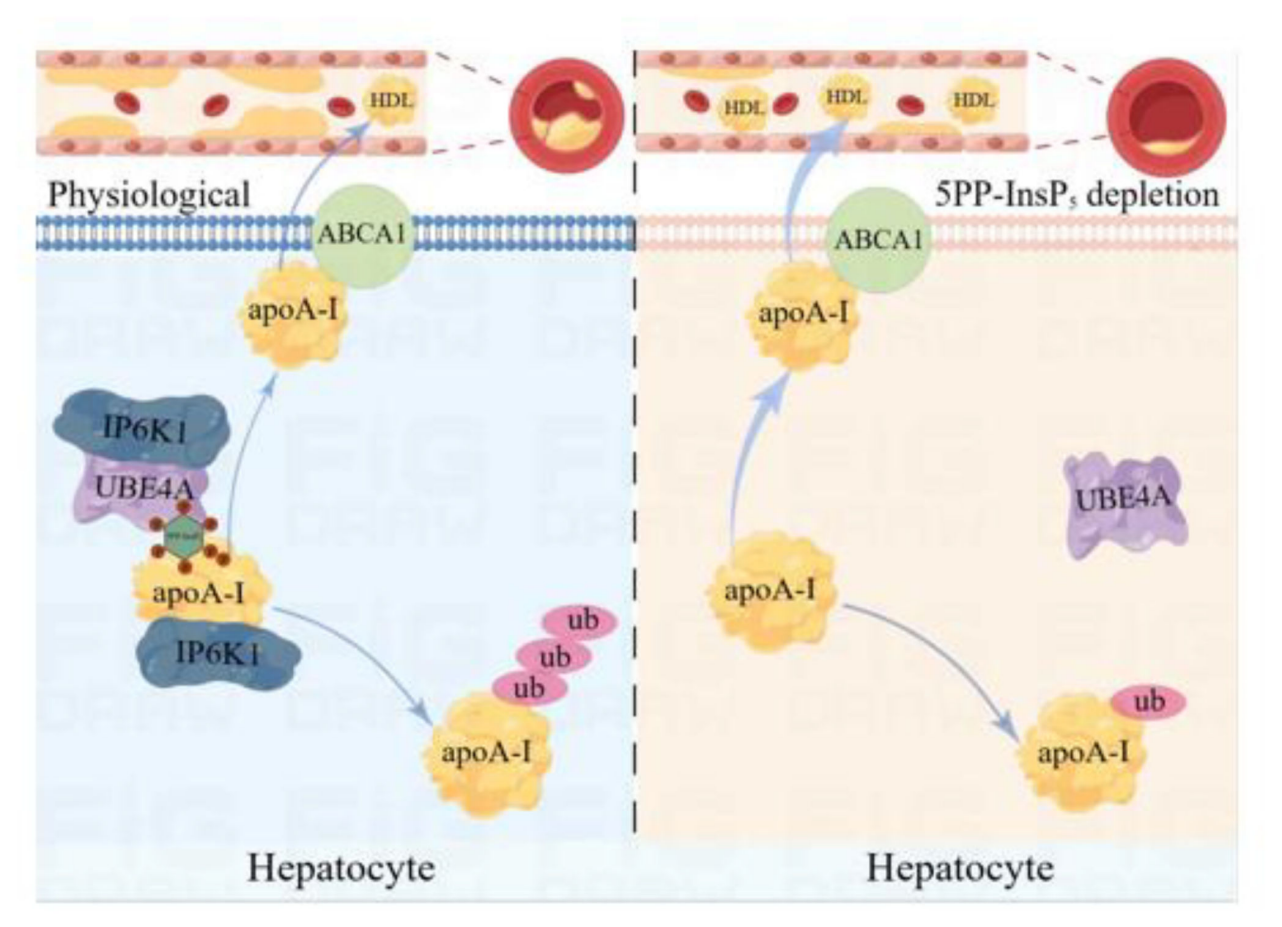
Model of 5PP-InsP_5_ depletion increasing apoA-I expression, augmenting reverse cholesterol transport, and attenuating atherosclerosis. **(Left)** IP6K1 physiologically binds to apoA-I and UBE4A. A local pool of 5PP-InsP_5_ was produced by IP6K1 to enhance the interaction of apoA-I with UBE4A. This leads to apoA-I ubiquitination and degradation. **(Right)** Depleting 5PP-InsP_5_ by genetic deletion or pharmacological inhibition of IP6K1 disrupts the binding of UBE4A to apoA-I. This allows apoA-I to escape from degradation. ABCA1 interacts with apoA-I to facilitate the formation of nascent HDL, which is then released into the plasma. The higher levels of apoA-I are linked to enhanced reverse cholesterol transport activity, which reduces atherosclerosis.

In this study, we demonstrate that genetic deletion or pharmacological inhibition of IP6K1 in hepatocytes upregulates apoA-I, a major structural and functional protein of HDL, augmenting cholesterol efflux, and attenuating atherosclerosis. IP6K1 binds to apoA-I in hepatocytes and generates 5PP-InsP_5_ to induce apoA-I degradation. In hepatocytes, IP6K1 also binds to UBE4A and recruits it to ubiquitinate apoA-I. The interaction of UBE4A with apoA-I is enhanced by 5PP-InsP_5_. Deleting IP6K1 or depleting 5PP-InsP_5_ reduces the binding of UBE4A to apoA-I, lowers apoA-I ubiquitination, and raises apoA-I protein levels. Our study demonstrates a mechanism by which depleting 5PP-InsP_5_ in hepatocytes boosts apoA-I production and thus increases cholesterol efflux, which can be amenable for attenuating atherosclerosis (Fig. 7).

Multiple lines of evidence support the concept that enhancing HDL-mediated reverse cholesterol transport is a promising antiatherogenic strategy[4, 5, 10, 12], Clinical studies reveal that it is the cholesterol efflux capacity rather than the HDL cholesterol level, inversely associated with the severity of atherosclerosis[43, 45]. ApoA-I is the major structural and functional protein of HDL. Although four weekly infusions of apoA-I could not prevent the recurrence of cardiovascular events after acute myocardial infarction through 90 days[46], intravenous administration of apoA-I increases cholesterol efflux capacity[47, 48], and displays a promising outcome in inducing regression of coronary atherosclerosis[12]. Increasing *de novo* biosynthesis of apoA-I from hepatocytes represents a practical strategy in treating atherosclerosis[10]. IP6K1 protein levels are increased in obesity[49], and patients with nonalcoholic fatty liver disease[39], which is associated with reduced plasma levels of apoA-I and HDL[50]. Our study demonstrates that depleting 5PP-InsP_5_ by inhibiting IP6K1 is an effective approach to enhance the production of apoA-I and consequently lowers the rate of atherosclerosis. IP6K2 and IP6K3 do not likely share the role of IP6K1 in regulating apoA-1 production, because IP6K1 is mainly found in the cytosol (Suppl. Fig. 19A), while IP6K2 is primarily localized to the nucleus (Suppl. Fig. 19B), and IP6K3 is not expressed in hepatocytes (Suppl. Fig. 20).

ABCA1 is essential for the biogenesis of nascent HDL particles[51]. The molecular mechanisms of apoA-I interaction with ABCA1 have been elusive. Previous studies demonstrate that apoA-I can stabilize ABCA1 in cell culture models[52], but the increases in ABCA1 protein are not found in apoA-I transgenic mice, which could be the result of some form of chronic adaptation[52]. Similarly, elevating apoA-I in transgenic mice does not influence ABCA1 and ABCG1 expression[10]. Consistently, our study shows that deletion of IP6K1 does not affect the protein levels of ABCA1 nor its interaction with apoA-I, indicating that IP6K1 does not affect the ABCA1-dependent biogenesis and release of nascent HDL particles. In line with the concept that IP6K1 and its product 5PP-InsP_5_ function in compartmentalized subcellular areas[15], IP6K1 binds to apoA-I in the endoplasmic reticulum. We speculate that this process occurs prior to apoA-I interaction with ABCA1 (Fig. 7), however, a comprehensive study is required to address this conjecture.

UBE4A is an E3 ubiquitin ligase that induces protein degradation[25]. We found that UBE4A can be recruited by IP6K1 via its product to induce apoA-I degradation. Targeting UBE4A has shown beneficial effects in some pathological conditions, such as inhibiting cancer cell proliferation by preventing UBE4A-mediated p53 degradation[23], and enhancing host antiviral responses by preventing UBE4A-mediated viperin degradation [24]. On the other hand, deleting UBE4A causes severe defects in animal models such as neuronal developmental defects[22], insulin resistance and hepatic steatosis [21], suggesting that UBE4A may not be a suitable treatment target for metabolic disease. In contrast, deletion of IP6K1 in hepatocytes enhances apoA-I production by preventing UBE4A-mediated apoA-I degradation, and subsequent attenuates atherosclerosis. Besides, deletion of IP6K1 increases insulin sensitivity[27], and alleviates hepatic metabolic dysfunction[39], indicating that IP6K1 may be a potential treatment target for metabolic diseases.

5PP-InsP_5_ can mediates protein degradation by directly binding to its target proteins[19] [40]. Besides, it can also pyrophosphorylate its target proteins[15, 53]. Pyrophosphorylation is not required in 5PP-InsP_5_-mediated binding of UBE4A to apoA-I, and this 5PP-InsP_5_-mediated binding of UBE4A to apoA-I was not solely due to negative charge, because 1PP-InsP_5_ and 3PP-InsP_5_ did not enhance the interaction. Furthermore, Ins(1,4,5)P_3_, Ins(1,3,4,5)P_4_ and Ins(1,3,4,5,6)P_5_ did not display similar effects to 5PP-InsP_5_ in promoting the binding of UBE4A to apoA-I.

Our study has several limitations. As females are less sensitive to the increase of apoA-I/HDL levels[54], male mice were utilized in this study. The effects of IP6K1 inhibition on atherosclerosis in females require a comprehensive study. Although cholesterol efflux plays a major role in preventing atherosclerosis, there are many other atheroprotective roles of apoA-I, such as anti-inflammation[55], and enhancing collecting lymphatic functions[56]. There is a possibility that atherosclerosis in the setting of apoA-I-deletion is too severe to observe an improvement with IP6K1 deletion, although the *in vivo* study suggests that apoA-I is the mediator. Besides HDL the VLDL/CM fractions were also elevated in the hepatocyte-specific *IP6K1* KO mice, indicating that deleting IP6K1 may also affect other factors that influence blood lipids in the hepatocytes, which warrants another study. InsP_8_ is generated by PPIP5Ks, and is a downstream metabolite of 5PP-InsP_5_. Our result does not exclude the possibility that InsP_8_ affects apoA-I protein levels, although neither deletion of PPIP5K1 nor PPIP5K2 affected the protein levels of apoA-I. It has been proposed that converting 5PP-InsP_5_ to InsP_8_ may serve as a mechanism to alleviate the impact of 5PP-InsP_5_[15]. Thus, the functional consequences of InsP_8_ upon apoA-I production warrant future investigation. IP6K1 has been recognized as a potential druggable target for diabetes. The signaling pathways of IP6K1 in regulating blood glucose and insulin sensitivity have been investigating by many groups[27, 57]. It is important to clarify that our study primarily focused on apoA-I, which plays a major role in preventing the development of atherosclerosis. Diabetes is also a risk factor for atherosclerosis. Future research into the role of IP6K1 in diabetes-related atherosclerosis will deepen our understanding in the pathological mechanisms of atherosclerosis.

## 5 Conclusions

Preclinical studies have demonstrated that knockout of IP6K1 improves insulin sensitivity, glucose tolerance and lowers myocardial ischemia-reperfusion injury [19, 27]. In this study, we found that deletion of IP6K1 attenuates atherosclerosis. Taken together, these pieces of evidence support IP6K1 as a potential therapeutic target for metabolic dysfunction and cardiovascular diseases[15]. IP6K1 might be a target for boosting plasma apoA-I to confer atheroprotection.

## Supplementary Material

supplementary information

## Data Availability

The authors declare that the data supporting the findings of this study are available in the paper and supplementary materials.

## Highlights

- IP6K1 binds to apoA-I and UBE4A in hepatocytes.
- IP6K1 via its product 5PP-InsP_5_ to enhance UBE4A-mediated apoA-I degradation.
- Hepatocyte specific deletion of IP6K1 elevates plasma levels of apoA-I.
- Hepatocyte specific deletion of IP6K1 enhances cholesterol efflux.
- Hepatocyte specific deletion of IP6K1 attenuates atherosclerosis.
