# Supplementary material for: Inhibiting IP6K1 confers atheroprotection by elevating circulating apolipoprotein A-I
Source: Metabolism. Author manuscript; Available in PMC 2024 Dec 16. (PMC7617243; doi:10.1016/j.metabol.2024.156098)
Supplement: supplementary information [file EMS201729-supplement-supplementary_information.pdf]

Supplementary Figures

Title： Inhibiting IP6K1 confers atheroprotection by elevating circulating apolipoprotein A-I

Authors: Xiaoqi Liu[1], Zixuan Zhang[1], Tim Aguirre[2], Megan L. Shipton[3], Lin Fu[4], Jimin Du[1], David Furkert[2], Ji Qi[5], Alfred C. Chin[6], Andrew M. Riley[3], Tong Liu[4], Xu Zhang[1], Barry V. L. Potter[3], Dorothea Fiedler[2], Yi Zhu[1,*], Chenglai Fu[1,5,*]

Author affiliations:

[1]Tianjin Key Laboratory of Metabolic Diseases, Department of Physiology and Pathophysiology, The Province and Ministry Co-Sponsored Collaborative Innovation Center for Medical Epigenetics, Tianjin Medical University, 22 Qixiangtai Road, Tianjin, 300070, China.

[2]Leibniz-Forschungsinstitut für Molekulare Pharmakologie (FMP), Berlin, 13125, Germany.

[3]Medicinal Chemistry & Drug Discovery, Department of Pharmacology, University of Oxford, Mansfield Road, Oxford OX1 3QT, UK.

[4]Tianjin Key Laboratory of Ionic-Molecular Function of Cardiovascular Disease, Department of Cardiology, Tianjin Institute of Cardiology, Second Hospital of Tianjin Medical University, Tianjin, 300211, China.

[5]Institute for Developmental and Regenerative Cardiovascular Medicine, Xinhua Hospital, School of Medicine, Shanghai Jiao Tong University, Shanghai 200092, China.

[6]Weill Cornell/Rockefeller/Sloan Kettering Tri-Institutional MD-PhD Program, New York, NY, 10065, USA.

*To whom correspondence should be addressed. Yi Zhu, email: zhuyi@tmu.edu.cn, or Chenglai Fu, email: fuchenglai@xinhuamed.com.cn.

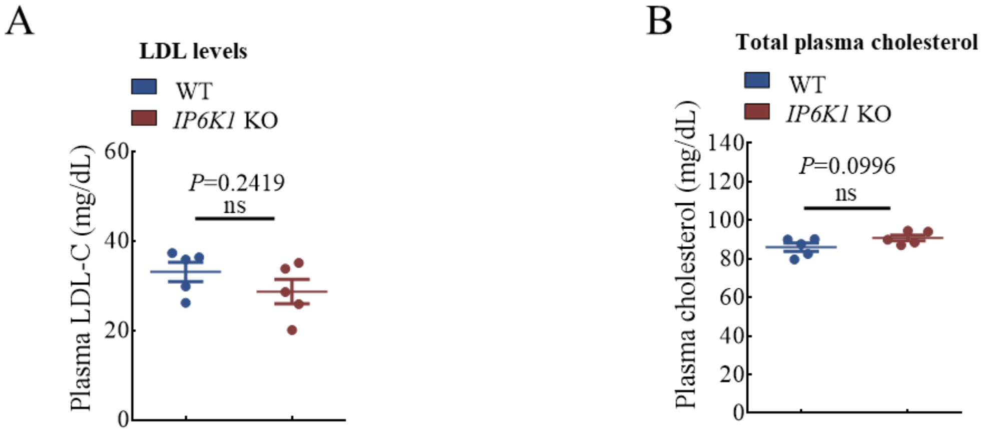

Supplementary Figure 1. Plasma LDL and total cholesterol levels in WT and *IP6K1* KO mice at baseline. A, Plasma LDL cholesterol levels in WT and *IP6K1* KO mice at baseline. Data represent mean±SEM, Student's *t*-test, n=5 mice per group. **B**, Plasma total cholesterol levels in WT and *IP6K1* KO mice at baseline. Data represent mean±SEM, Student's *t*-test, n=5 mice per group.

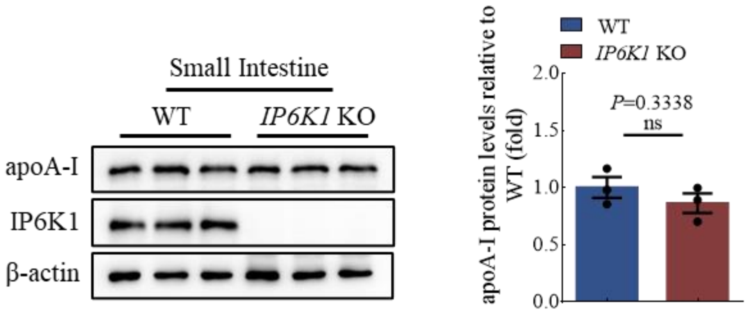

Supplementary Figure 2. Deletion of IP6K1 did not augment apoA-I protein levels in the small intestines. Protein levels of apoA-I in the small intestines were similar between WT and *IP6K1* KOs. Data represent mean±SEM, Student's *t*-test, n=3 mice per group.

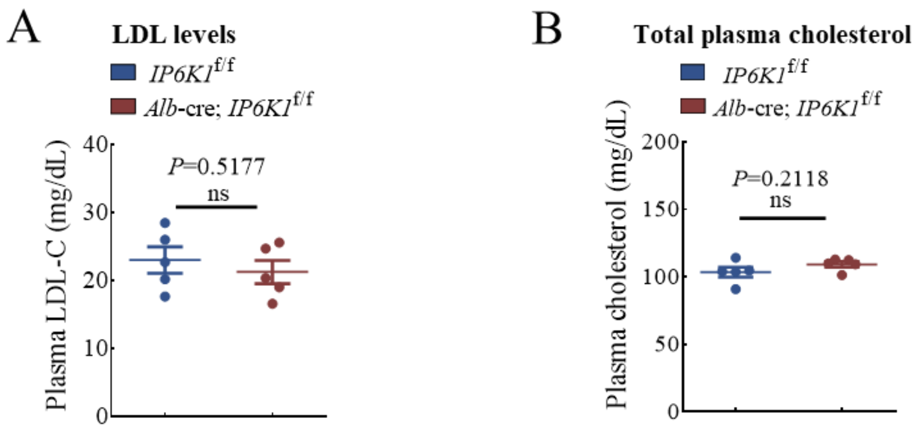

Supplementary Figure 3. Plasma LDL and total cholesterol levels in hepatocyte-specific *IP6K1* KO (*Alb*-cre; *IP6K1*$^{f/f}$) and control (*IP6K1*$^{f/f}$) mice at baseline. A, Plasma LDL cholesterol levels in hepatocyte-specific *IP6K1* KO (*Alb*-cre; *IP6K1*$^{f/f}$) and control (*IP6K1*$^{f/f}$) mice at baseline. Data represent mean±SEM, Student's *t*-test, n=5 mice per group. **B**, Plasma total cholesterol levels in hepatocyte-specific *IP6K1* KO (*Alb*-cre; *IP6K1*$^{f/f}$) and control (*IP6K1*$^{f/f}$) mice at baseline. Data represent mean±SEM, Student's *t*-test, n=5 mice per group.

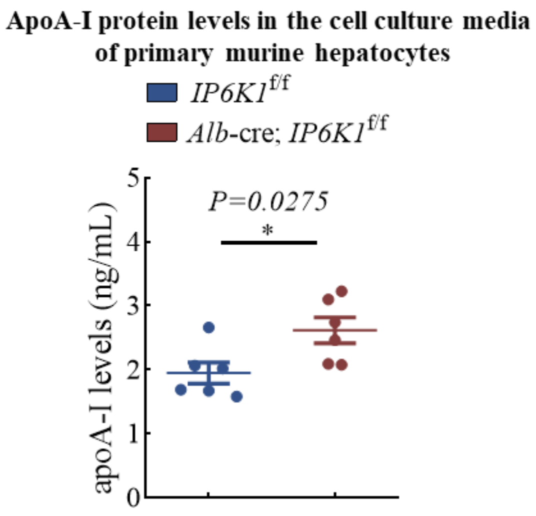

Supplementary Figure 4. The protein levels of apoA-I in the cell culture media were higher in the *IP6K1* KO hepatocytes preparations. The primary murine hepatocytes of hepatocyte-specific *IP6K1* KO (*Alb*-cre; *IP6K1*$^{f/f}$) and control (*IP6K1*$^{f/f}$) mice were isolated and cultured *in vitro*. The cells were culture in a serum-free medium for 24 h. The cell culture media were then collected, and the apoA-I protein levels in the cell culture media were determined by ELISA assay. Data represent mean±SEM, Student's *t*-test, n=6 mice per group.

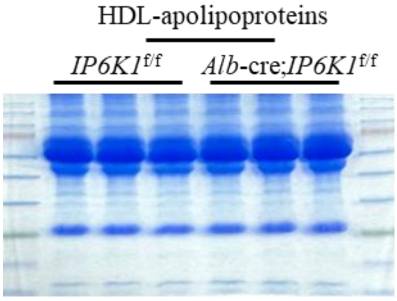

Supplementary Figure 5. Deletion of IP6K1 did not affect the protein components of HDL. HDL was purified from the plasma of hepatocyte-specific *IP6K1* KO (*Alb*-cre; *IP6K1*$^{f/f}$) and control (*IP6K1*$^{f/f}$) mice. The HDL proteins were separated by SDS PAGE and visualized by Coomassie blue staining. n=3 mice per group.

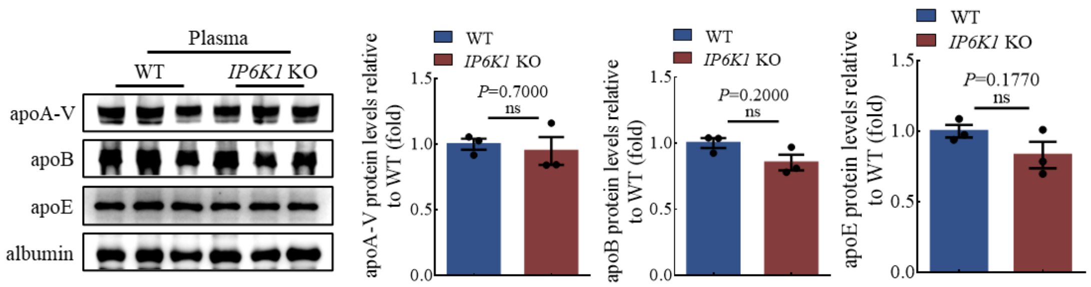

Supplementary Figure 6. Deletion of IP6K1 did not affect the plasma levels of apoA-V, apoB, or apoE. Western blots showed that the protein levels of apoA-V, apoB, and apoE in the plasma were not significantly different between WT and *IP6K1* KOs. Data represent mean±SEM, n=3 mice per group, Mann-Whitney U test for apoA-V and apoB. Student's *t*-test for apoE.

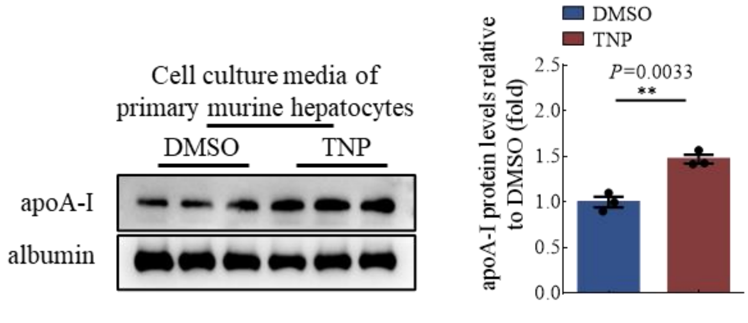

Supplementary Figure 7. Inhibiting IP6K activity by TNP treatment elevated the protein levels of apoA-I in the cell culture media. Primary murine hepatocytes were treated with TNP (3 μM) for 24 hours. The protein levels of apoA-I in the cell culture media were higher in the TNP-treated preparations. Data represent mean±SEM, Student's *t*-test, n=3 independent repeats.

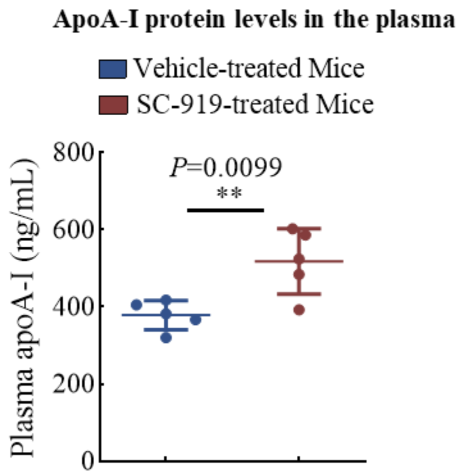

Supplementary Figure 8. Blocking IP6K activity by SC-919 increased plasma levels of apoA-I. WT Mice were administered with SC-919 (10 mg/kg) or vehicle control for one week. The protein levels of apoA-I in the plasma were determined by ELISA assay. Data represent mean±SEM, Student's *t*-test, n=5 mice per group.

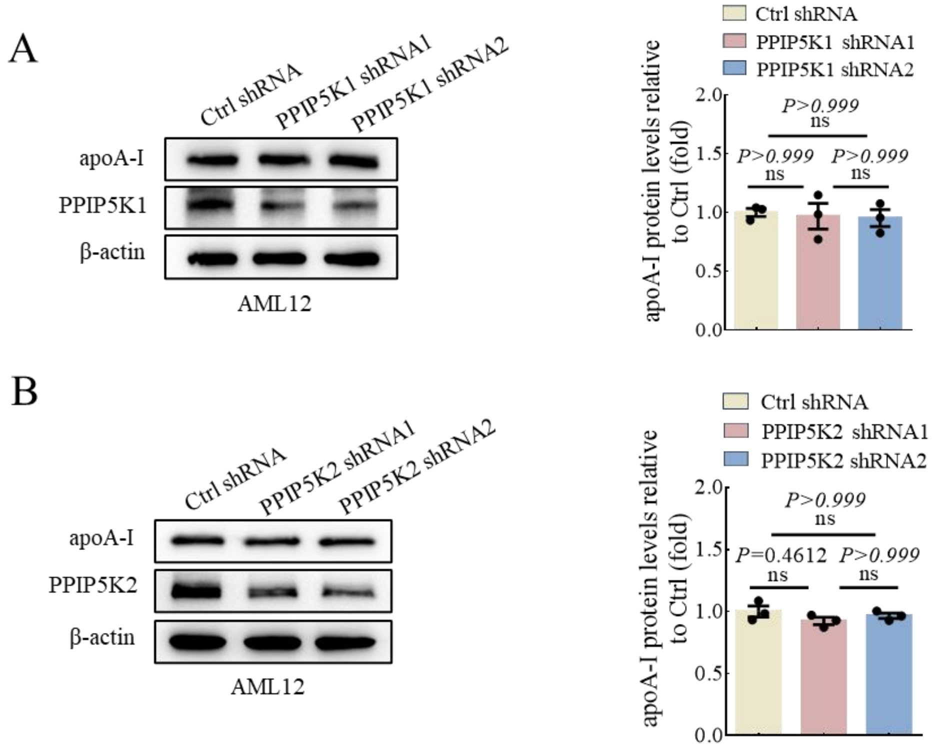

Supplementary Figure 9. Deletion of PPIP5K1 or PPIP5K2 in murine hepatocyte AML 12 cells did not affect the expression levels of apoA-I. A, Deleting PPIP5K1 in AML12 hepatocytes by shRNA transduction did not affect the protein levels of apoA-I. Data represent mean±SEM, One-way ANOVA, n=3 independent repeats. **B**, Deleting PPIP5K2 in AML12 hepatocytes by shRNA transduction did not affect the protein levels of apoA-I. Data represent mean±SEM, One-way ANOVA, n=3 independent repeats.

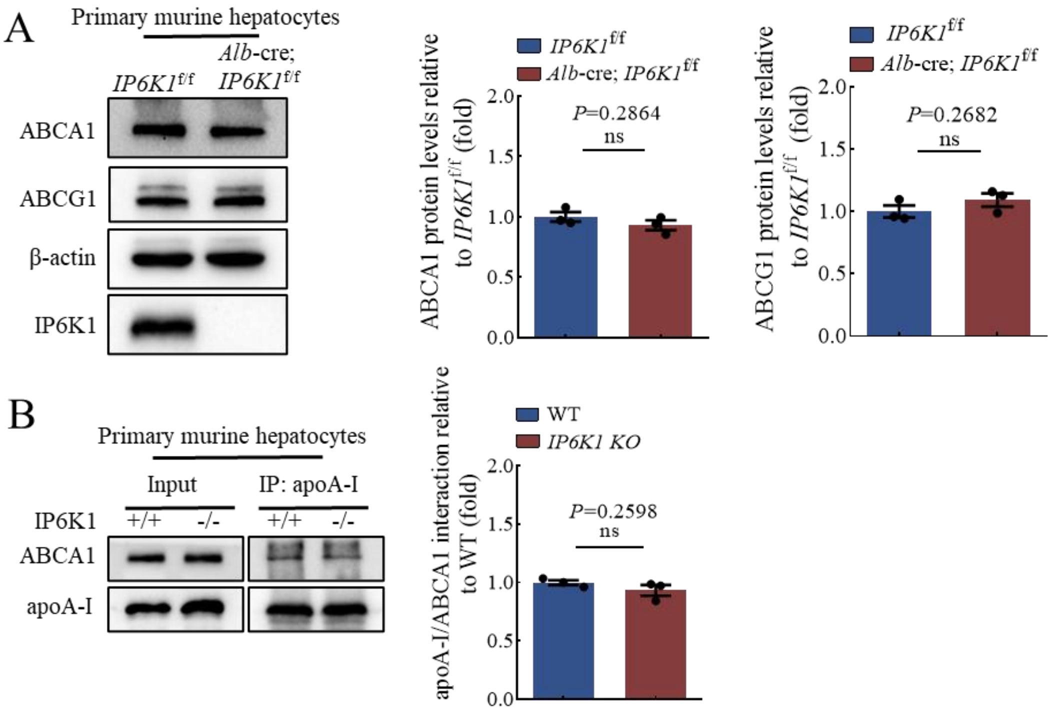

Supplementary Figure 10. Deletion of IP6K1 did not affect the protein levels of ABCA1 and ABCG1 in hepatocytes and did not affect the interaction of apoA-I with ABCA1. A, Protein levels of ABCA1 and ABCG1 in WT and *IP6K1* KO primary murine hepatocytes were similar. Data represent mean±SEM, Student's *t*-test, n=3 independent repeats. **B**, Immunoprecipitations of apoA-I co-pulled down equal amounts of ABCA1 in WT and *IP6K1* KO primary murine hepatocytes. Data represent mean±SEM, Mann-Whitney U test, n=3 independent repeats.

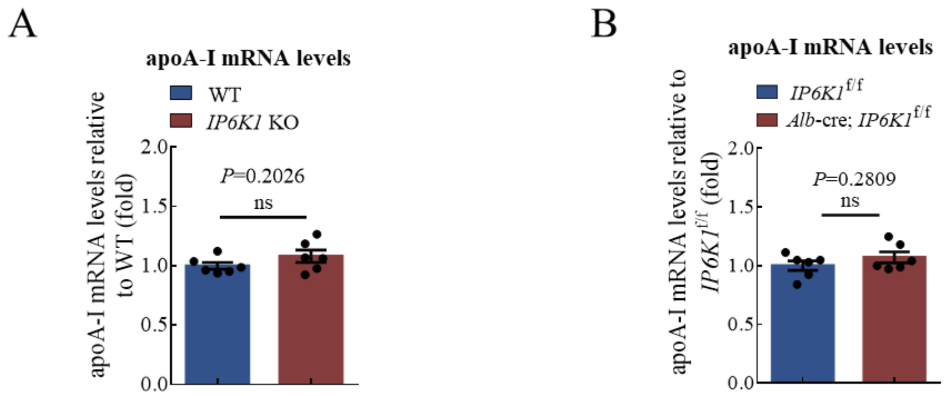

Supplementary Figure 11. Deletion of IP6K1 did not affect apoA-I mRNA levels.

A, ApoA-I mRNA levels in WT and *IP6K1* KO primary murine hepatocytes were similar. Data represent mean±SEM, Student's *t*-test, n=6 independent repeats. **B**, ApoA-I mRNA levels in primary murine hepatocytes of hepatocyte-specific *IP6K1* KOs (*Alb*-cre; *IP6K1*$^{f/f}$) and controls (*IP6K1*$^{f/f}$) and were similar. Data represent mean±SEM, Student's *t*-test, n=6 independent repeats.

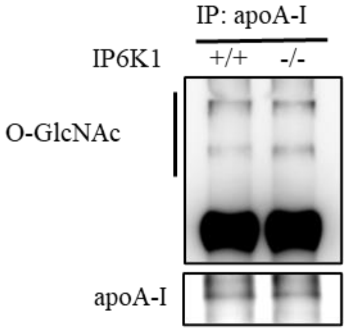

Supplementary Figure 12. IP6K1 did not regulate apoA-I by affecting its O-GlcNAcylation levels in hepatocytes. ApoA-I was immunoprecipitated in primary murine hepatocytes of WT and *IP6K1* KOs and blotted for O-GlcNAc.

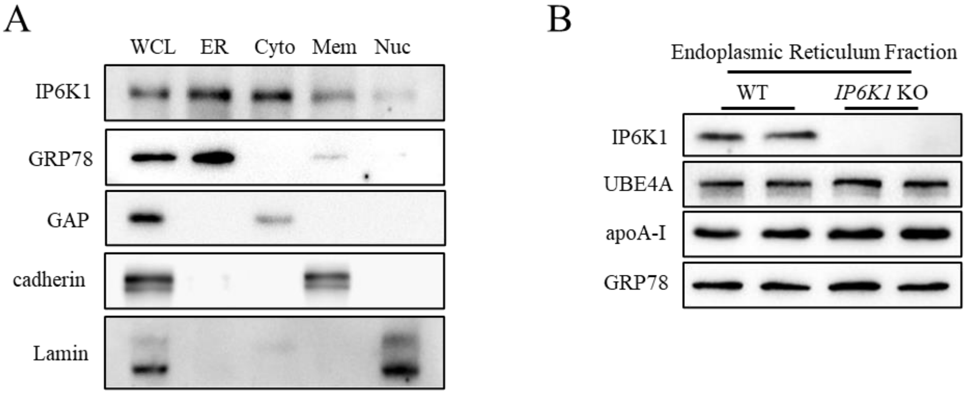

Supplementary Figure 13. IP6K1 was in the endoplasmic reticulum. A, Subcellular fractions of primary murine hepatocytes were isolated. Western blots showed that IP6K1 was in the endoplasmic reticulum fractions. WCL, whole cell lysates. ER, endoplasmic reticulum. Cyto, cytosol. Mem, membrane. Nuc, nucleus. **B**, Endoplasmic reticulum of WT and *IP6K1* KO hepatocytes were isolated. Western blots showed that IP6K1 was in the WT but not the *IP6K1* KO preparations.

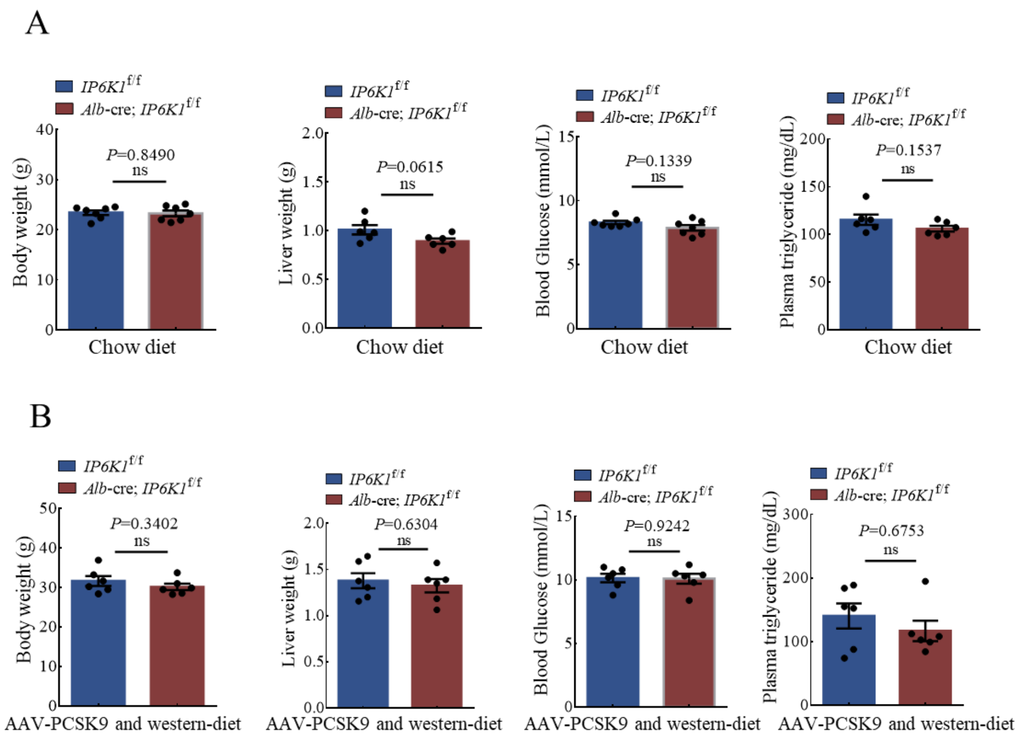

Supplementary Figure 14. The body weights, liver weights, blood glucose levels, and plasma triglyceride levels of hepatocyte-specific *IP6K1* KO (*Alb*-cre; *IP6K1*$^{f/f}$) and control (*IP6K1*$^{f/f}$) mice were similar. A, Body weights, liver weights, blood glucose levels, and plasma triglyceride levels of hepatocyte-specific *IP6K1* KO (*Alb*-cre; *IP6K1*$^{f/f}$) and control (*IP6K1*$^{f/f}$) mice were not significantly different at baseline. Data represent mean±SEM, Student's *t*-test, n=6 mice per group. **B**, Animals were injected with AAV-PCSK9 and fed a Western diet. The body weights (Student's *t*-test), liver weights (Student's *t*-test), blood glucose levels (Student's *t*-test), and plasma triglyceride levels (Mann-Whitney U test) of hepatocyte-specific *IP6K1* KO (*Alb*-cre; *IP6K1*$^{f/f}$) and control (*IP6K1*$^{f/f}$) mice were not significantly different. Data represent mean±SEM, n=6 mice per group.

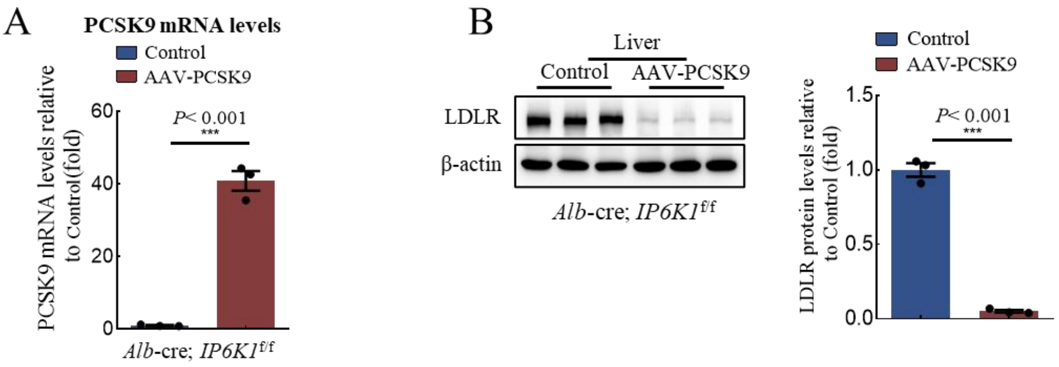

Supplementary Figure 15. Injection of AAV-PCSK9 increased the expression levels of PCSK9 and decreased the protein levels of LDLR in the livers. Mice were injected with AAV-PCSK9 and injected with PBS as a control. **A**, Real-time PCR results showed that the mRNA levels of PCSK9 in the livers were higher in the AAV-PCSK9-treated group. Data represent mean±SEM, Student's *t*-test, n=3 mice per group. **B**, Protein levels of LDLR in the livers were lower in the AAV-PCSK9-treated group. Data represent mean±SEM, Student's *t*-test, n=3 mice per group.

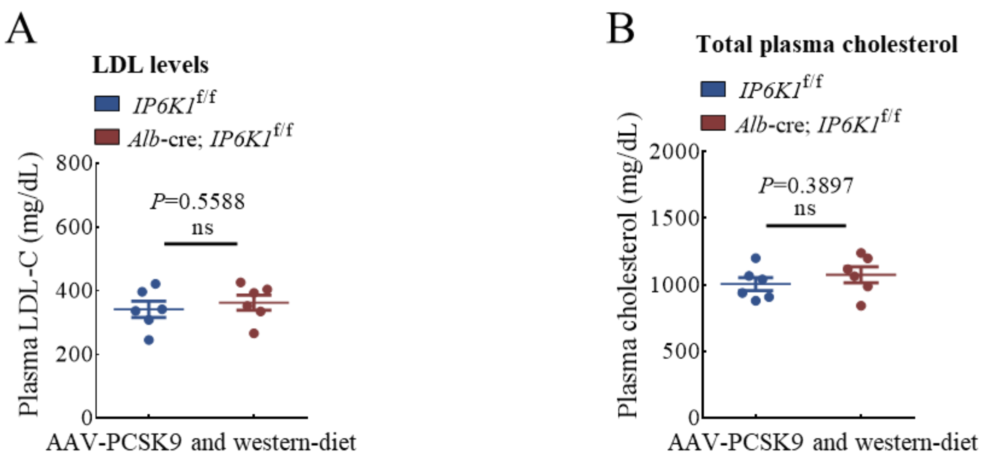

Supplementary Figure 16. Plasma LDL and total cholesterol levels in the AAV-PCSK9 and Western diet-treated hepatocyte-specific *IP6K1* KO (*Alb*-cre; *IP6K1*$^{f/f}$) and control (*IP6K1*$^{f/f}$) mice. A, Plasma LDL cholesterol levels in the AAV-PCSK9 and Western diet-treated hepatocyte-specific *IP6K1* KO (*Alb*-cre; *IP6K1*$^{f/f}$) and control (*IP6K1*$^{f/f}$) mice. Data represent mean±SEM, Student's *t*-test, n=6 mice per group. **B**, Plasma total cholesterol levels in the AAV-PCSK9 and Western diet-treated hepatocyte-specific *IP6K1* KO (*Alb*-cre; *IP6K1*$^{f/f}$) and control (*IP6K1*$^{f/f}$) mice. Data represent mean±SEM, Student's *t*-test, n=6 mice per group.

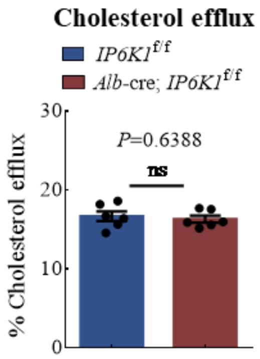

Equal amounts of apoA-I from control (*IP6K1*$^{f/f}$) and hepatocyte-specific *IP6K1* KO (*Alb*-cre; *IP6K1*$^{f/f}$) hepatocytes were added to the assay

Supplementary Figure 17. The apoA-I from hepatocyte-specific *IP6K1* KO (*Alb*-cre; *IP6K1*$^{f/f}$) and control (*IP6K1*$^{f/f}$) hepatocytes displayed similar activity in cellular cholesterol efflux assay. ApoA-I expressed in hepatocyte-specific *IP6K1* KO (*Alb*-cre; *IP6K1*$^{f/f}$) and control (*IP6K1*$^{f/f}$) hepatocytes were purified and applied to the cellular cholesterol efflux assay. Data represent mean±SEM, Student's *t*-test, n=6 independent repeats.

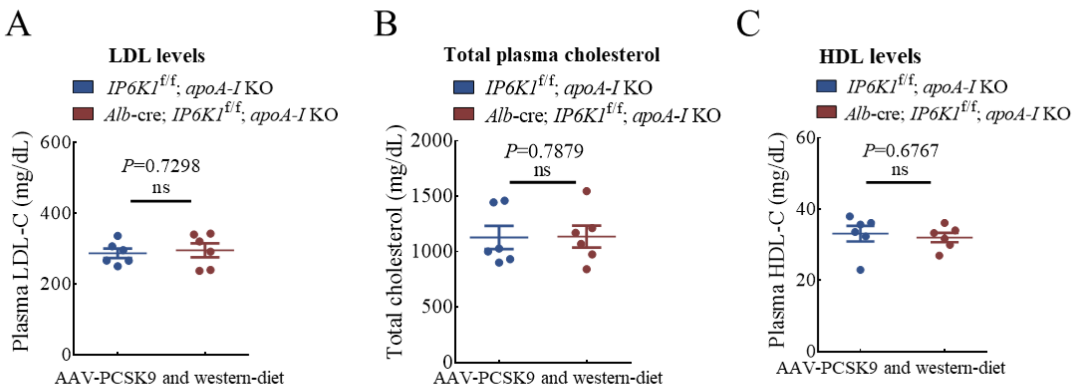

Supplementary Figure 18. Plasma LDL, total cholesterol, and HDL levels in the AAV-PCSK9 and Western diet-treated *IP6K1*$^{f/f}$; *apoA-I* KO and *Alb*-cre; *IP6K1*$^{f/f}$; *apoA-I* KO mice. A, Plasma LDL levels were similar between the AAV-PCSK9 and Western diet-treated *IP6K1*$^{f/f}$; *apoA-I* KO and *Alb*-cre; *IP6K1*$^{f/f}$; *apoA-I* KO mice. Data represent mean±SEM, Student's *t*-test, n=6 mice per group. **B**, Total plasma cholesterol levels were similar between the AAV-PCSK9 and Western diet-treated *IP6K1*$^{f/f}$; *apoA-I* KO and *Alb*-cre; *IP6K1*$^{f/f}$; *apoA-I* KO mice. Data represent mean±SEM, Mann-Whitney U test, n=6 mice per group. **C**, Plasma HDL levels were similar between the AAV-PCSK9 and Western diet-treated *IP6K1*$^{f/f}$; *apoA-I* KO and *Alb*-cre; *IP6K1*$^{f/f}$; *apoA-I* KO mice. Data represent mean±SEM, Student's *t*-test, n=6 mice per group.

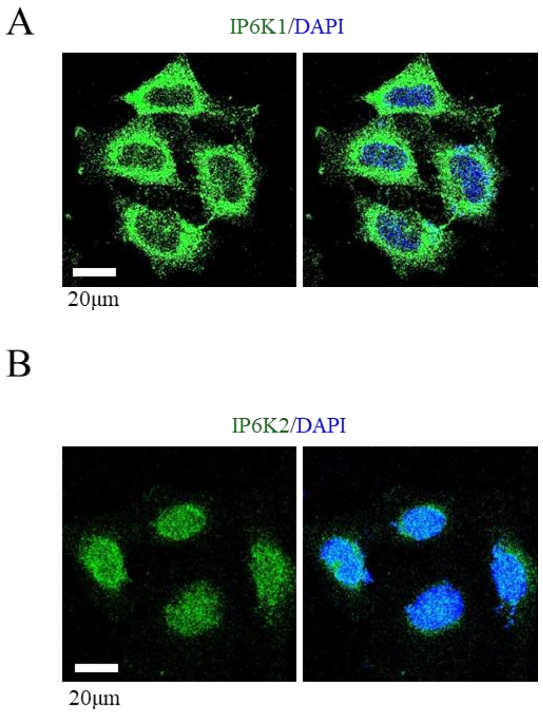

Supplementary Figure 19. Subcellular localization of IP6K1 and IP6K2 in hepatocytes. **A**, Immunostaining of IP6K1 in primary murine hepatocytes. The nuclei were stained by DAPI. Scale bar 20μm. **B**, Immunostaining of IP6K2 in primary murine hepatocytes. The nuclei were stained by DAPI. Scale bar 20μm.

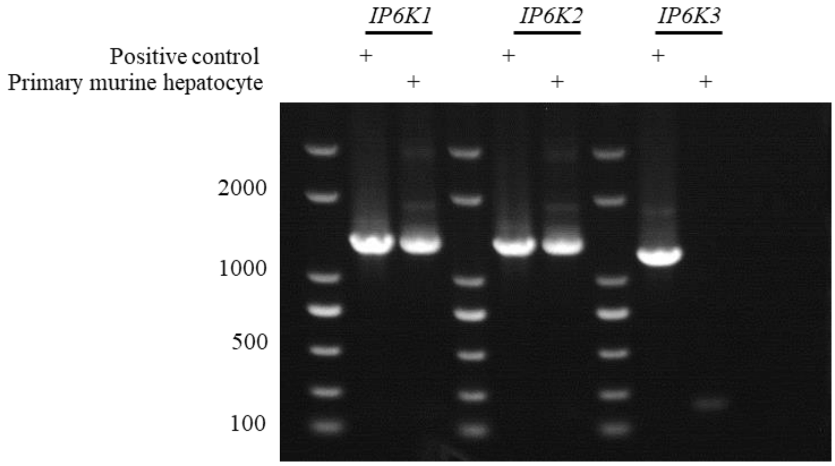

Supplementary Figure 20. IP6K3 was not expressed in primary murine hepatocytes. Messenger RNA from primary murine hepatocytes was reverse-transcribed to cDNA. RT-PCR results showed that IP6K1 and IP6K2 were expressed, whereas IP6K3 was not expressed in primary murine hepatocytes. cDNA from the mouse brain was used as the positive control.
